# Synthetic Cell Armor Made of DNA Origami

**DOI:** 10.1021/acs.nanolett.3c01878

**Published:** 2023-07-18

**Authors:** Weitao Wang, Peter R. Hayes, Xi Ren, Rebecca E. Taylor

**Affiliations:** † Department of Mechanical Engineering, Carnegie Mellon University, Pittsburgh, Pennsylvania 15213, United States; ‡ Department of Chemical Engineering, Carnegie Mellon University, Pittsburgh, Pennsylvania 15213, United States; ¶ Department of Biomedical Engineering, Carnegie Mellon University, Pittsburgh, Pennsylvania 15213, United States; § Department of Electrical and Computer Engineering, Carnegie Mellon University, Pittsburgh, Pennsylvania 15213, United States

**Keywords:** cellular plasma membrane, DNA origami, cell encapsulation, membrane biophysics, cell manipulation

## Abstract

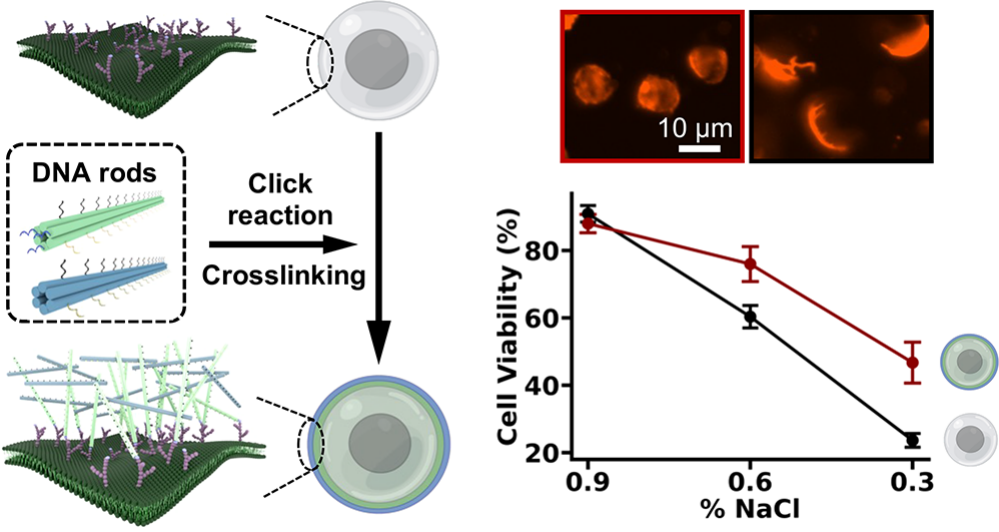

The bioengineering applications of cells, such as cell printing and multicellular assembly, are directly limited by cell damage and death due to a harsh environment. Improved cellular robustness thus motivates investigations into cell encapsulation, which provides essential protection. Here we target the cell-surface glycocalyx and cross-link two layers of DNA nanorods on the cellular plasma membrane to form a modular and programmable nanoshell. We show that the DNA origami nanoshell modulates the biophysical properties of cell membranes by enhancing the membrane stiffness and lowering the lipid fluidity. The nanoshell also serves as armor to protect cells and improve their viability against mechanical stress from osmotic imbalance, centrifugal forces, and fluid shear stress. Moreover, it enables mediated cell–cell interactions for effective and robust multicellular assembly. Our results demonstrate the potential of the nanoshell, not only as a cellular protection strategy but also as a platform for cell and cell membrane manipulation.

The cellular plasma membrane serves as a protective barrier by encapsulating cellular components.^1−3^ This biomembrane is decorated with membrane-bound proteins, making it essential for mediating cellular signaling and sensing.^1−4^ The plasma membrane is also linked to the interior cytoskeleton that mechanically supports the cell to maintain its size, shape, and integrity.^5,6^ It therefore allows for cellular communication while shielding the cell from outside assaults. However, the cell membrane is often unable to protect the cell from external stressors, for example, the high forces and subsequent large membrane deformations experienced during cell manipulation and delivery applications in tissue engineering and regenerative medicine.^7−9^

Cell encapsulation is recognized as one approach to tackle this problem with various nanomaterials being extensively investigated to wrap the whole cell for cellular protection and manipulation.^10−15^ However, the lack of material programmability limits control over the encapsulation, such as the tunability of the encapsulation formation and its on-demand removal. Moreover, material overload as well as the cytotoxic nature of certain materials may hinder cell function and even lead to cell death.^16−19^ Recently, structural DNA nanotechnology including DNA origami has emerged as a powerful tool for studies interfacing cell biology with engineered nanostructures.^20−26^ Multiple studies have utilized DNA as a building block for cell encapsulations.^12−15^ For example, using hybrid chain reaction (HCR) and polymerization, cross-linking networks are constructed on cell membranes for cell protection and the manipulation of cell–cell interactions.^12,14,15^ While these approaches have successfully achieved biocompatible and effective cell encapsulation, demonstrating promising potential in cell delivery, manipulation, and identification, there has been limited exploration of the impact of encapsulation coating on cell membranes. For example, previous studies have applied DNA nanostructures to coat cells for membrane deformation and sculpting.^27−30^ However, the success of such approaches has primarily been demonstrated on artificial lipid bilayers. The effects of the DNA nanostructure coating on live cell membranes, particularly in terms of membrane mechanics, remain largely unexplored. Furthermore, the reconfiguration and polymerization of membrane-coated DNA origami on giant unilamellar vesicles (GUVs) can influence the coating pattern and distribution, highlighting the dynamic interactions between the membrane and membrane-bound assemblies.^27,30−32^ Yet, live cell membranes are mechanically supported and protected by the cytoskeleton and complex membrane-bound protein networks. It remains unknown whether they will exhibit reactions similar to those of DNA origami-coated GUVs, necessitating further investigation. Thus, a nanoshell approach utilizing DNA origami technique offers unique opportunity to enable stable and modular cell encapsulation and potentially address these questions with excellent design capability, high specificity and programmability.^22,25^

In this work, we describe a nanoshell encapsulation strategy that targets the cell-surface glycocalyx, which utilizes two layers of DNA nanorods by sequentially recruiting and cross-linking them onto cell membranes under physiological conditions. We demonstrated the modularity and tunability of the nanoshell by varying the layering and composition of the DNA nanorods. We further investigated the impact of the nanoshell on the biophysical properties of our cell-nanoshell systems by examining cell membrane biomechanics, membrane lipid fluidity, and the distribution and morphology of DNA constructs after anchoring onto the membrane. Moreover, we showcased the protective effects of the nanoshell via enhanced viability under three environmental stressors: osmotic swelling, centrifugation, and fluid shear stress. Further, we demonstrated that the nanoshell enabled effective and robust multicellular assembly through mediated cell–cell interactions. With this encapsulation strategy, we can build nanoshells potentially not only for cellular protection and manipulation but also as a tool for modulating membrane biomechanics and exploring the effects of these changes on cell behavior and function.

The nanoshell is designed to consist of two layers of cross-linking DNA nanorods that we referred to as rod A and rod B (both ∼7 nm in diameter and ∼400 nm in length). The rods were decorated with multiple functional ssDNA binding overhangs (Figure 1A and Supplementary Figure 1). Specifically, rod A had three anchoring ssDNA (a-ssDNA) for allowing the anchoring of rod A to membrane glycocalyx-anchored a-ssDNA complementary initiators (a′-ssDNA), 14 uniformly distributed staining ssDNA (s-ssDNA) for biotin attachment and subsequent streptavidin-fluorophore staining, and 14 uniformly distributed hybridization ssDNA (h-ssDNA) for the cross binding of rod A and rod B through ssDNA hybridization. Rod B nanostructures had 14 s-ssDNA and 14 hybridization ssDNA complementary (h′-ssDNA) that allowed them to bind to h-ssDNA-decorated rod A. The numbers of h-ssDNA and h'-ssDNA were optimized to promote rods hybridization. Further increases in quantity led to unstable rod formation (Supplementary Figure 2). An atomic force microscopy (AFM) image verified the formation of the rods, which were constructed as six-helical bundles (Figure 1A). Gel electrophoresis confirmed the monodispersity of the individual DNA origami rods. When combined, the formation of an aggregate indicates the successful binding of the two rod species by hybridization (Figure 1B). Two other distinct bands suggest that rod mononers and dimers existed at the same time. As the nanorods will be used for cell culture applications, we further examined their stabilities in a cell culture medium (Figure 1C). Individual rods had minimal degradation after 6 and 30 h incubation at 37 °C in both cell culture medium. The aggregate, formed by the combination of rod A and B, did not degrade noticeably for up to 30 h of incubation as well.

**Figure 1:**
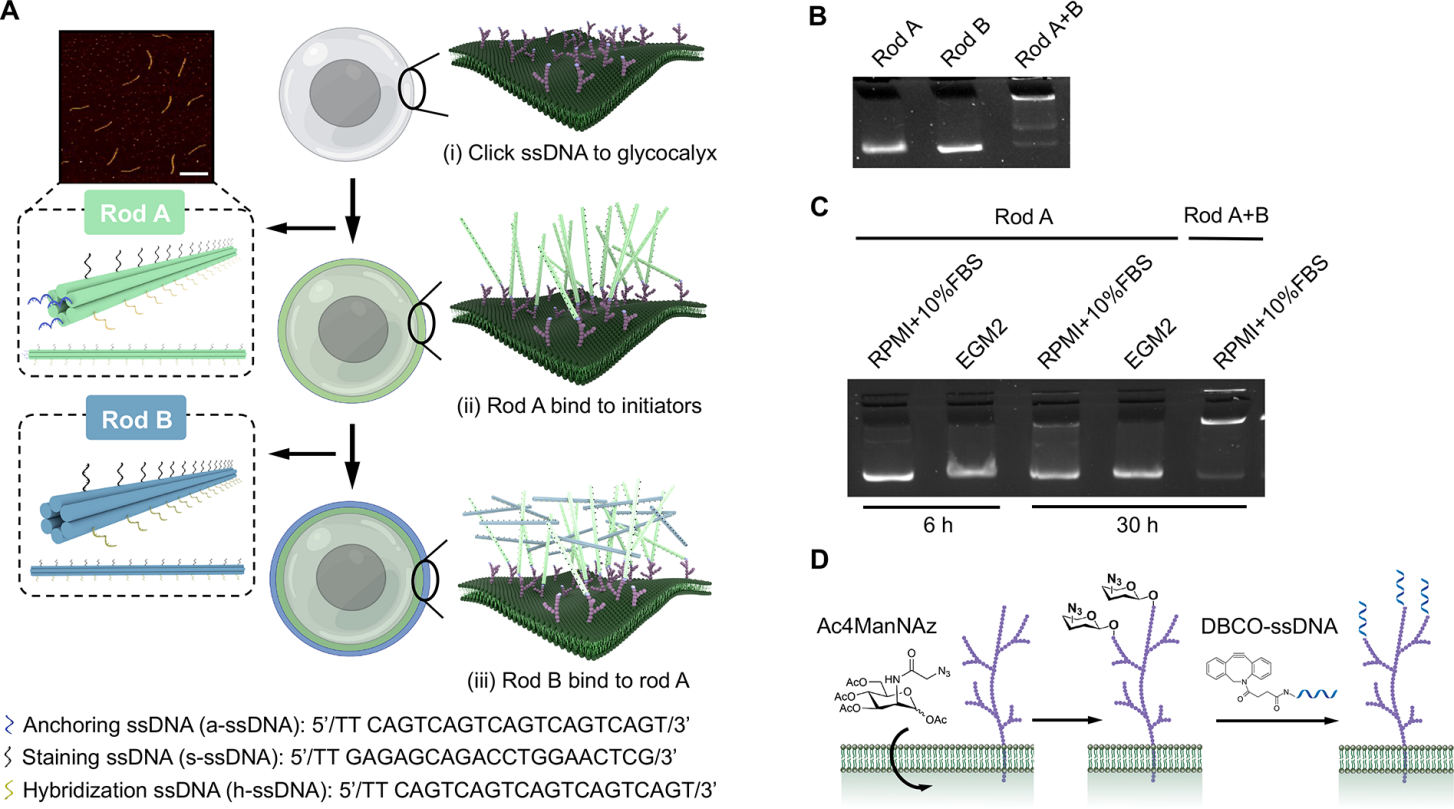
Schematics of the nanoshell synthesis process and validation of stability. (A) Synthesis of the DNA nanoshell on the cellular membrane through a three-step immobilization process including: (i) the immobilization of a′-ssDNA initiators on the glycocalyx; (ii) the binding of rod A (green) to a′-ssDNA via ssDNA hybridization, and (iii) the binding and cross-linking of rod B (blue) to rod A via the hybridization of h-ssDNA on rod A and h′-ssDNA on rod B. AFM imaging of rods was shown. Both rod A and B were ∼7 nm in diameter and ∼400 nm in length. Three a-ssDNA (blue), 14 s-ssDNA (black), and 14 h-ssDNA (yellow) were uniformly distributed on rod A. Fourteen s-ssDNA (black) and 14 h′-ssDNA (yellow) were uniformly distributed on rod B. All ssDNA overhangs were 22 base pairs. Scale bar: 500 nm. (B) Agarose gel electrophoresis of individual DNA rods and a mixture of rods after 30 min incubation of rod A and rod B at 37 °C. (C) Agarose gel electrophoresis studies of the stability of individual DNA rods and the aggregate in two types of cell culture medium. Rod A and rods mixtures were incubated at 37 °C for 6 and 30 h in each cell culture medium. (D) The immobilization of DBCO-labeled a′-ssDNA initiators on azide-presenting cell-surface glycocalyx through copper free click chemistry.

To anchor rod A to the plasma membrane, using Jurkat cells as a suspended mammalian cell model, we utilized a method we have previously reported to first immobilize a′-ssDNA initiators onto the cell-surface glycocalyx (Figure 1D).^33^ In this method, azide ligands were covalently incorporated onto glycocalyx through metabolic glycan labeling using an azido monosaccharide, *N*-azidoacetylmannosamine-tetraacylated (Ac4ManNAz). a′-ssDNA were conjugated with dibenzocyclooctyne (DBCO) to form DBCO-a′-ssDNA through an NHS-Ester and amine reaction. Bioorthogonal glycocalyx labeling with copper-free click chemistry allowed the conjugation of azide ligands on glycocalyx and DBCO-a′-ssDNA, leading to the immobilization of a′-ssDNA on glycocalyx. For these studies, rod A was first introduced for glycocalyx binding, followed by rod B for hybridization to rod A.

We observed the successful recruitment of rod A to the membrane and rod B to rod A using fluorescence microscopy (Figure 2A). Confocal microscopy cross-sectional images of cells coated with both rods and fluorescence intensity profiles extracted from those images further confirmed both binding events and the formation of a nanoshell structure (Figure 2B). To confirm the efficacy of this approach on more than one mammalian cell type, we replicated the synthesis strategy on human umbilical vein endothelial cells (HUVECs), demonstrating the versatility and utility of this nanoshell encapsulation technique for both nonadherent and adherent cell types (Supplementary Figure 3). As the glycocalyx is on the surface of almost every mammalian cell, we expect this glycocalyx-targeting method to be applicable to a broad array of cell types.^34,35^

**Figure 2:**
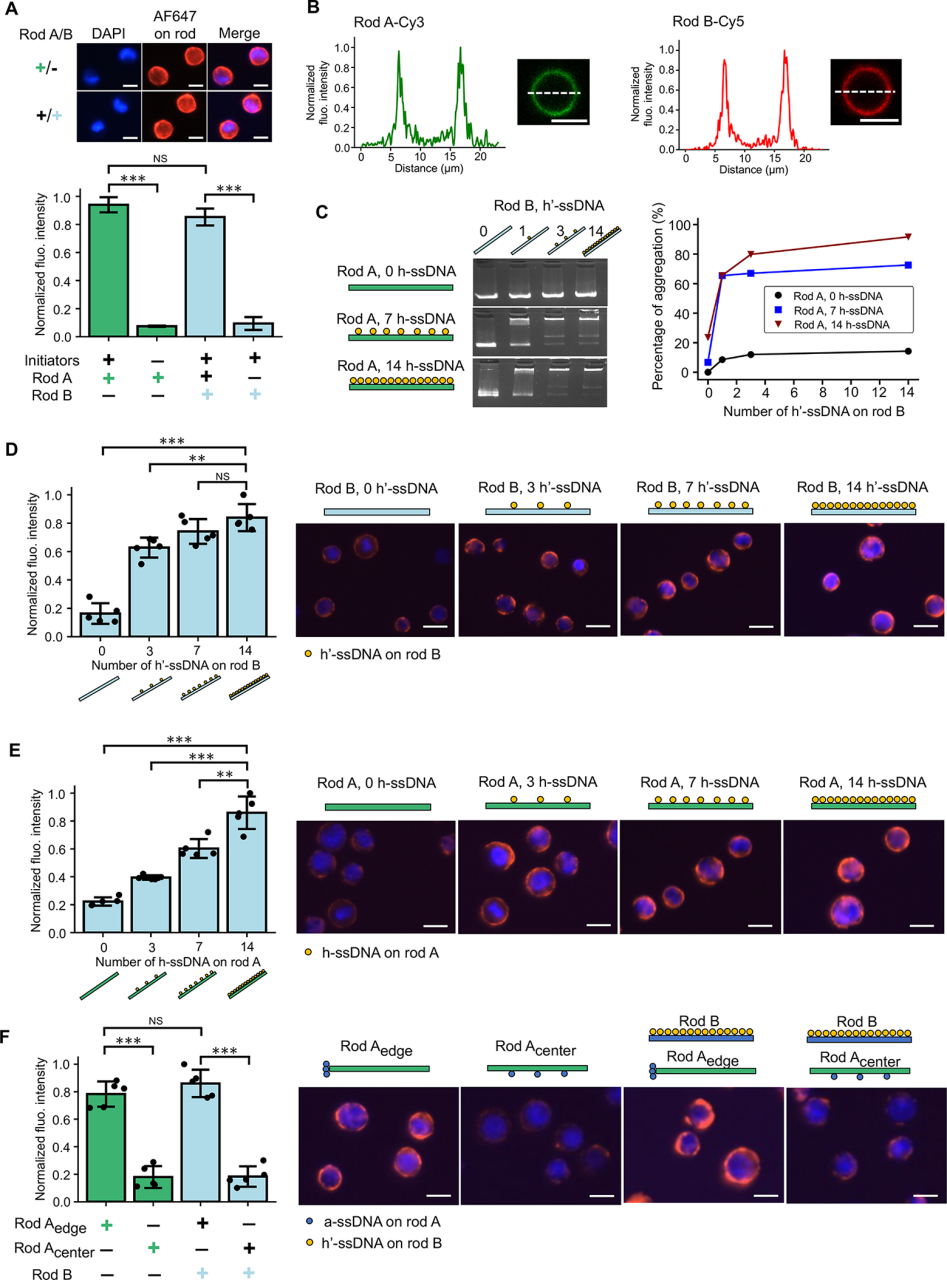
Modulation of the DNA nanoshell composition by engineering the position and number of overhangs on rods. (A) Fluorescence imaging and intensity quantification of four experimental conditions including (i) rod A (biotin) coating with a′-ssDNA initiators on the glycocalyx, (ii) rod A (biotin) coating without a′-ssDNA initiators, (iii) rod B (biotin) coating with both the initiators and rod A, and (iv) rod B (biotin) coating with initiators but without rod A. Biotin-labeled rods were stained with streptavidin-AF647 (red). Cell nuclei were stained with DAPI (blue). The green and blue ± symbols and accompanying bar graphs indicated whether rod A or rod B populations, respectively, were fluorescently labeled. (B) Normalized fluorescence intensity profiles of nanoshell-coated cells with Cy3-staining rod A (green) and Cy5-staining rod B (red). (C) Gel electrophoresis studies for the quantification of the percentage of aggegate resulting from each mixture of DNA rods A and B as a function of the number of hybridization overhangs on rods. The number of h-ssDNA on rod A was changed from 0 to 7 to 14 and the number of h′-ssDNA on rod B was changed from 0 to 1 to 3 to 14. (D) Modulating the binding of rod B to rod A by changing the number of h′-ssDNA on rod B. The number of h′-ssDNA was changed from 0 to 3 to 7 to 14. Rod A with 14 h-ssDNA was used in this experiment. Rod B was stained with AF647 (red). (E) Modulating the binding of rod B to rod A by changing the number of h-ssDNA on rod A. The number of h-ssDNA was changed from 0 to 3 to 7 to 14. Rod B with 14 h′-ssDNA was used in this experiment. Rod B was stained with AF647 (red). (F) Modulating the binding of rod A onto the cell membrane and rod B to rod A by changing the position of a-ssDNA on rod A. The fluorescence intensity quantification of four conditions includes (i) edge-decorated rod A (biotin), (ii) center-decorated rod A (biotin), (iii) edge-decorated rod A and rod B (biotin), and (iv) center-decorated rod A and rod B (biotin). Biotin-labeled rods were stained with streptavidin-AF647 (red). Schematics of rods and representative fluorescence microscope images were shown. Yellow and blue circles in (C)–(F) represented h/h′-ssDNA on both rods and a-ssDNA on rod A, respectively. Data were presented as means ± s.d. in (A), (D), (E), and (F). **P ≤ 0.01, ***P ≤ 0.001. All scale bars: 10 μm.

To demonstrate our ability to engineer the nanoshell, we systematically probed the roles of functional ssDNA binding overhangs extending from the DNA nanorods, investigating how the multivalency and positions of the overhangs modulate the amount of rod binding in the nanoshell. First, we performed gel electrophoresis studies by mixing DNA rod A and B with varying numbers of h-ssDNA and h′-ssDNA in suspension and incubating for 0.5 h at 37 °C to allow for hybridization. The number of evenly displayed h-ssDNA on rod A was modified from 0 to 7 to 14, and the number of h′-ssDNA on rod B was from 0 to 1 to 3 to 14. Gel electrophoresis images and the quantification showed a monotonic decline of single rod band intensity and a monotonic increase in the aggregate band intensity with increasing number of h-ssDNA and h′-ssDNA (Figure 2C). To confirm this finding on Jurkat cells, we first labeled the cells with rod A bearing 14 h-ssDNA overhangs. Next, we introduced fluorescently labeled rod B with 0, 3, 7, and 14 h′-ssDNA. We found the binding of rod B increased monotonically again with increasing number of h′-ssDNA on rod B (Figure 2D). In addition, fluorescently labeled rod B with 14 h′-ssDNA was introduced to bind to rod A with 0, 3, 7, and 14 h-ssDNA. A similar trend of an increasing amount of rod B binding with increasing valency of h-ssDNA on rod A was observed (Figure 2E). Furthermore, the binding of rods was also increased by adding higher concentrations of rods (Supplementary Figure 4).

While we learned that the multivalency of hybridization ssDNA on rods A and B regulated the recruitment of rod B onto rod A, we also found the position of anchoring ssDNA on rod A to be critical for the recruitment of both rods onto cell membranes. Rod A was modified to display a-ssDNA in two configurations: at the edge and at the center (Figure 2F). The binding of edge-decorated rod A to the glycocalyx and subsequent binding of rod B to rod A were significantly more than that of center-decorated rod A. This finding is consistent with previous studies, stating that the recruitment of DNA nanostructures presenting ssDNA overhangs at the sharp or “pointy” areas is more efficient.^26,36,37^ Our results demonstrate our ability to modulate the number of nanorods incorporated into the nanoshell by changing the valency and positioning of functional ssDNA overhangs on both rods. As the maximum thickness of the nanoshell is defined by the length of rod A, an increase in rod binding indicates a higher density of rods. As a result of these findings, all following studies were performed with three edge-located a-ssDNA and 14 side-located h-ssDNA on rod A and 14 side-located h′-ssDNA on rod B, unless otherwise stated.

Cells constantly internalize substances outside the membrane, inducing membrane remodeling and deformations at multiple scales.^38,39^ It is therefore important to evaluate the stability of the nanoshell on the membrane, which will be particularly instructive for future DNA nanostructure-based biomedical applications, for example, the longer-term presentation of functional nanodevices and biomolecules on nanoshells. We first evaluated the surface retention time of the nanorods on the cell membrane as an indicator of stability. Nanoshell-coated cells and rod A-coated cells were incubated under three conditions: (i) 4 °C for 0.5 h, (ii) 37 °C for 0.5 h, and (iii) 37 °C for 3 h. The first incubation condition was regarded as a baseline, as membrane movements and cell activities, especially cellular uptake, which lead to the destabilization of the nanoshells, were minimal. For consistent comparison, only biotinylated-rod A was stained with streptavidin-AF647 and they were stained after incubation. This staining method allowed us to quantify only the rods remaining on the external cell surface. From fluorescence-activated cell sorting (FACS) data, the rod fluorescence intensity had a dramatic drop at 0.5 h incubation at 37 °C as compared to 4 °C, suggesting that single DNA rods had low stability after being anchored on cell membrane under physiological conditions (Figure 3A). The fluorescence signal continued to decrease with further incubation at 37 °C for 3 h, though at a slower rate. In contrast, on nanoshell-coated cells, we observed only a minimal decrease in fluorescence signal intensity with incubation at 37 °C, even after 3 h (Figure 3A). Next, we performed an examination of the cellular uptake of rod A for rod A-coated cells and nanoshell-coated cells using human umbilical vein endothelial cells (HUVECs). We found significantly more internalization of rod A on rod A-coated cells comparing to that of nanoshell-coated cells (Figure 3B). The improved surface retention time and decreased cellular uptake of rods showed that the cross-linking nanoshell had a higher stability and remained on the cell membrane for a longer duration, as compared to single rod attachment without cross-linking. Moreover, although having high stability under physiological conditions, the nanoshell can still be degraded through the simple administration of DNase I, making temporary encapsulation possible (Supplementary Figure 5). This feature is particularly important for applications requiring on-demand release or reconfiguration of the nanoshell.

**Figure 3:**
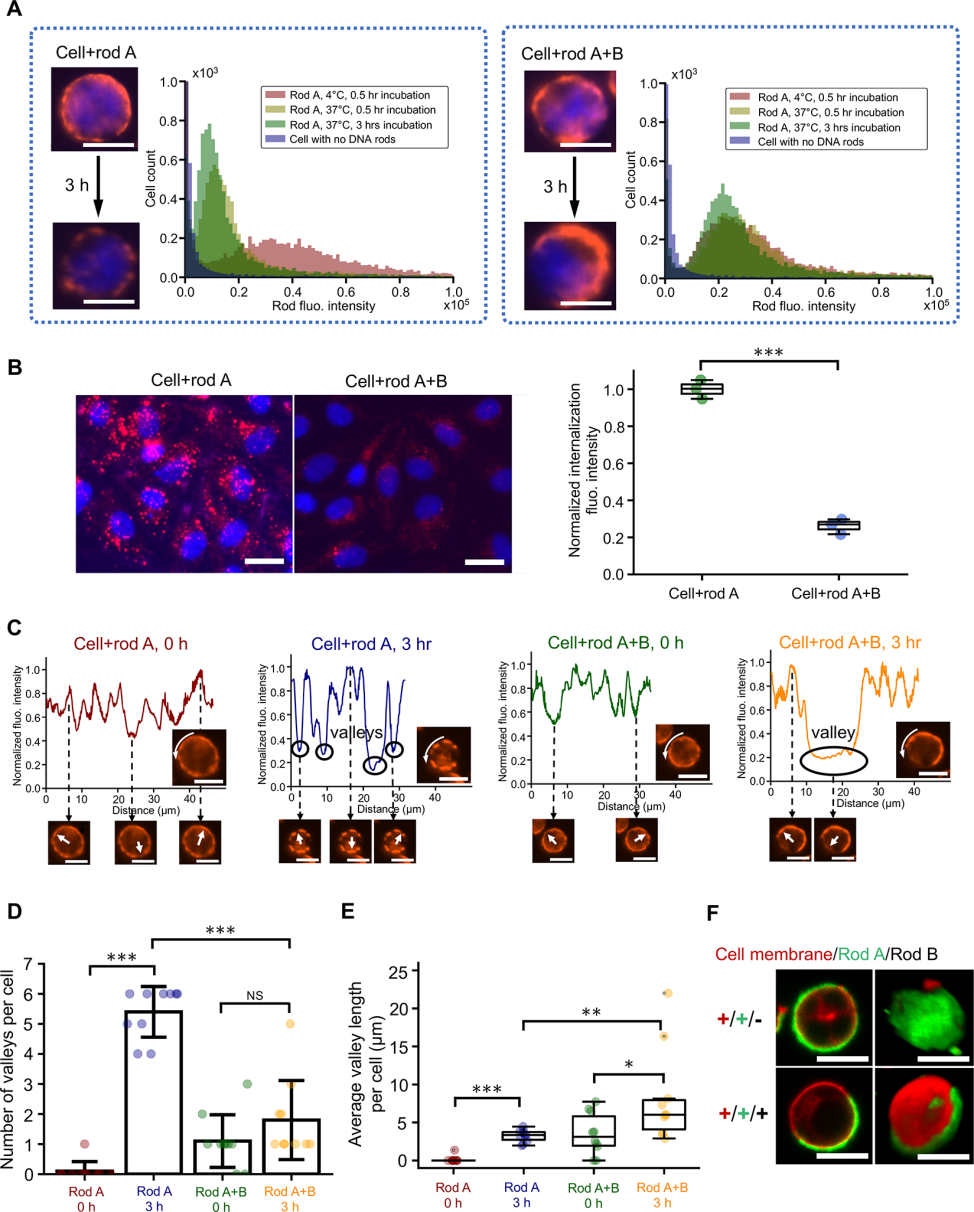
Cell surface retention time, cellular internalization, and morphology of the DNA nanoshell. (A) Cell surface retention time of DNA rods measured by fluorescence-activated cell sorting (FACS). Rod A-coated cells and nanoshell-coated cells were incubated at 4 °C for 0.5 h and 37 °C for both 0.5 and 3 h. The distribution of cell populations versus AF647 fluorescence intensity were plotted. Representative wide-field fluorescence microscope images were taken. Pretreatment native cells without fluorophore staining were used as the control. DNA rod A was stained with streptavidin-AF647 (red). Cell nuclei were stained with DAPI (blue). (B) Fluorescence imaging and quantification on the cellular uptake of DNA rod A on rod A-coated cells and nanoshell-coated cells using HUVECs. Red fluorescence signals represent internalized rod A stained with streptavidin-AF647. (C) Analysis of DNA rod distribution on the borders of cell membranes. The fluorescence intensity along the cell borders was tracked and plotted for rod A-coated cells and nanoshell-coated cells after 0 and 3 h incubation. DNA rod A was stained with AF647 (red). Wide-field fluorescence microscopy images showed the distribution of fluorescence signal on cells. Signal valleys were shown and marked in the plots. Signal valley quantification was reported in terms of (D) the number and (E) the length of fluorescence signal valleys in each group. *n* = 10. *P ≤ 0.05, **P ≤ 0.01, ***P ≤ 0.001. (F) Confocal microscopy cross-section images and reconstructed 3D models of representative rod A-coated cells and nanoshell-coated cells. DNA rod A was stained with Alexa fluor 488 (green). Cell membranes were stained with the DiD lipid dye (red). All scale bars: 10 μm.

Next, we sought to understand whether the DNA rods and nanoshells interact with the membrane after the rods are anchored onto the membrane. Noticeably, we observed substantial remodeling of nanoshell as the pattern of rod fluorescence signal evolved throughout incubation period (cell fluorescence images in Figure 3A). We tracked the distribution of fluorescence signals in cell images from these studies and measured the signal intensity around the contours of cell borders (Figure 3C). At the 0 h time point where the cellular uptake and membrane detachment of rods were minimal due to insufficient incubation time, relatively continuous and uniform intensity was observed in rod A-coated cells. Interestingly, however, the signal became discretized and nonuniform after 3 h incubation with signal intensity disappearing in discrete regions on cell borders, which resulted in signal valleys, suggesting long-term incubation destabilized the membrane-anchored DNA rods. Signal valleys also appeared in nanoshell-coated cells but were fewer in number and were substantially wider, potentially due to the cross-linking and polymerization of rods. We then quantified the number and length of signal valleys (Figure 3D,E). Data showed that the discretization of fluorescence signals was dependent on two factors: incubation time and the addition of cross-linking rod B. Incubation-induced signal valleys were presumably due to cellular uptake whereas cross-linking-induced valleys suggested that the rods remodeled their positions on the cell membrane during cross-linking process and incubation. A similar phenomenon was also reported previously on GUVs where the distribution of DNA origami and the morphology of lipid bilayers were altered after the triggered polymerization of DNA origami.^30,31^ We further imaged rod A-coated cells and nanoshell-coated cells with confocal microscopy and reconstructed their three-dimensional models. The images revealed uniform covering on rod A-coated cells and partial, localized coverage on nanoshell-coated cells (Figure 3F). Our findings showcase the important role of the dynamic interactions between the DNA rods and the cell membrane in repositioning membrane-anchored substances. However, in this study, we did not observe membrane deformations due to DNA construct polymerization, which has been reported in the previous literature.^27,30,31^

After demonstrating the ability of the nanoshell to remodel and stabilize itself, we sought to investigate whether this stabilization affects the biophysical properties of the cell membrane with a focus on membrane stiffness and lipid fluidity. First, membrane elastic modulus was evaluated by performing micropipette aspiration on pretreatment native cells, rod A-coated cells and nanoshell-coated cells (Figure 4A). Cells (*R* ≈ 10 μm) were aspirated into micropipettes (*R*_p_ = 2.5 μm) through aspiration pressure change Δ*P*. The elastic modulus *E* can be derived from the following equation, assuming the cell as a continuum-medium model with homogeneity,
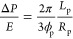
1where *L*_p_ is the aspiration length and ϕ_p_ ≈ 2.1.^40,41^ The membrane elastic modulus of nanoshell-coated cells was 0.340 ± 0.062 kPa (mean ± sd), which was around 3-fold that of native cells, measured to be 0.122 ± 0.029 kPa (Figure 4B,C). Interestingly, single rod A coating resulted in an increase in the membrane stiffness as well. We also observed a gradual increase in the elastic modulus with an increasing valency of h/h′-ssDNA, showing the tunability of the membrane stiffening. Our results indicate that the nanoshell formed by cross-linking rods mechanically supported the membrane and enhanced the membrane mechanics, functioning analogously to the cytoskeleton underneath the membrane.

**Figure 4:**
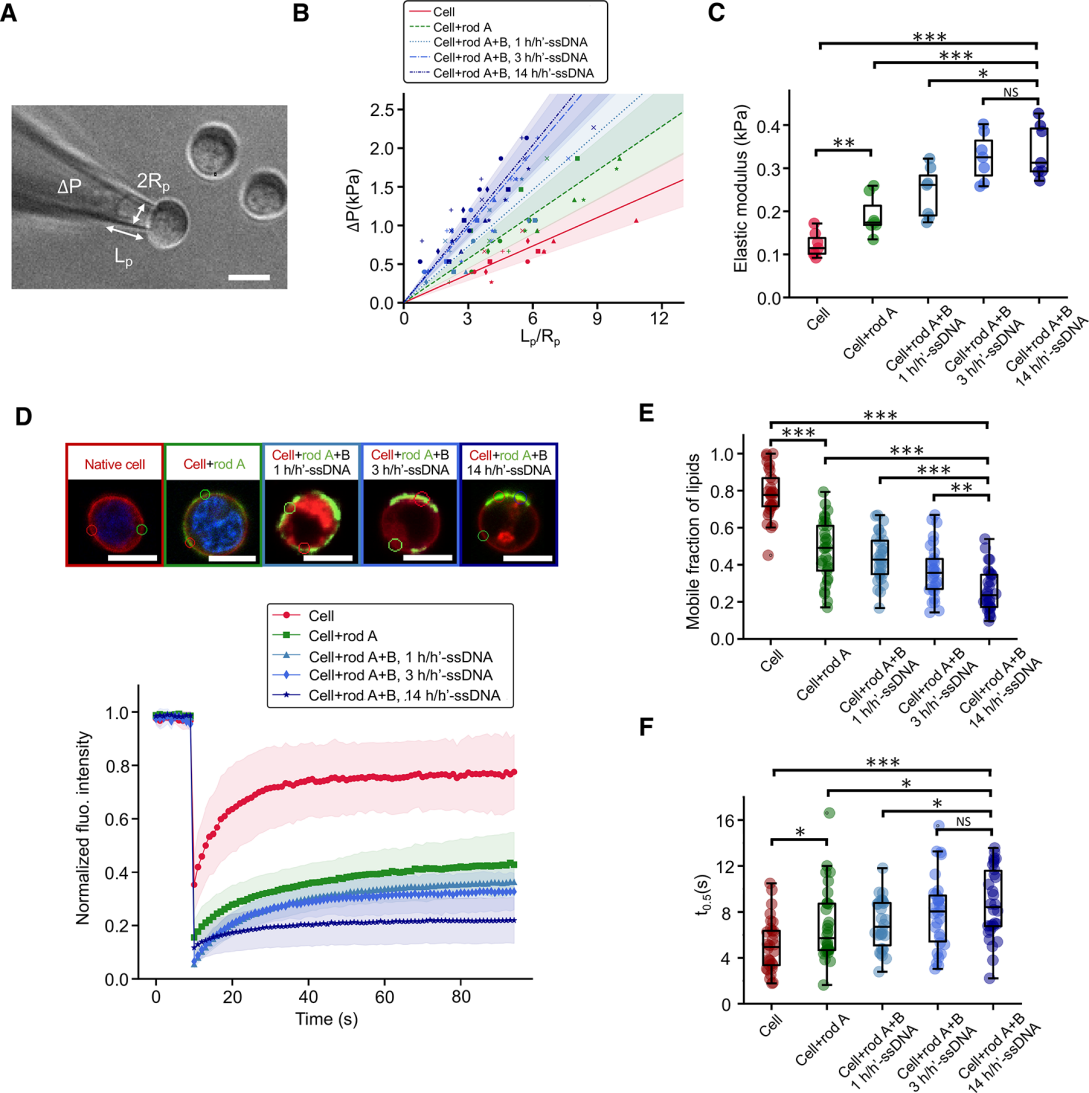
Tunable modulation of the biophysical properties of cell membranes by the DNA origami nanoshell. (A) A representative image of micropipet aspiration with a cell being aspirated into a micropipet with an inner radius *R*_p_ = 2.5 μm under an aspiration pressure Δ*P*. (B) Relationship between the Δ*P* and the normalized aspiration length . Colored data points represented cells including native cells, rod A-coated cells, and nanoshell-coated cells. Nanoshell-coated cells had rods with 1, 3, and 14 h/h′-ssDNA, respectively. (C) Evaluation of membrane elastic modulus.^40^*n* = 7 for (B) and (C). (D) Fluorescence recovery after photobleaching (FRAP) experiments and the normalized membrane lipid fluorescence intensity over time after photobleaching a region of interest in the membranes of cells with different rod coatings. Data were presented as means (solid lines) ± standard deviation (shadow areas) (*n* = 10). Cell membranes were stained with DiD lipid dye (red). DNA rod A were stained with streptavidin-AF488 (green). The color of the text represented fluorescence colors. The color of rectangles represented line colors in the plot. Evaluation of (E) of the mobile fraction of membrane lipid and (F) half-time recovery of membrane lipid of native cells, rod A-coated cells, and nanoshell-coated cells (rods with 1, 3, and 14 h/h′-ssDNA, respectively). *n* = 30 for (E) and (F). * *P* ≤ 0.05, ***P ≤ 0.001. All scale bars: 10 μm.

Given the global mechanical impact of the nanoshell on cell membrane mechanics, we sought to determine if the nanoshell could induce local changes to the fluidity of the membrane lipid. A previous study found that by decorating DNA origami on GUVs with cholesterol anchors, the fluidity of the artificial lipid was not affected.^28^ To address this question, we performed fluorescence recovery after photobleaching (FRAP) experiments to evaluate the mobile fraction of membrane lipid and the rate of recovery (Figure 4D). Interestingly, we observed that our glycocalyx-anchored nanoshell greatly reduced the lipid mobility (Figure 4E). Specifically, in native cells, fluorescence signal recovered to ∼80% of the prephotobleaching level within 30 s. However, for rod A-coated cells, the recovery dropped to ∼40%. The number further decreased when the two-component nanoshell was applied and when the valency of h/h′-ssDNA increased. The recovery here represents the mobile fraction of the membrane lipid. We also noticed that the rate of recovery was much slower after cells were coated with DNA rods (Figure 4F). The half-time recovery of the nanoshell-coated cell membrane lipid was only around half of that of the native cell membrane lipid. These results demonstrate that a certain amount of membrane lipid may experience gelation when the membrane was coated with DNA rods and nanoshell, especially the latter case, resulting in a significant decrease in membrane lipid mobility. Consistent with tunable membrane stiffening, the lipid fluidity also demonstrated tunability by altering the valency of overhangs, as more h/h′-ssDNA resulted in more rod cross-linking (Figure 4D–F). Intriguingly, the membrane-anchored DNA rods also lost their mobility, whereas by comparison, DBCO-cy5 had nearly the same mobility as membrane lipid (Supplementary Figure 6). Taken together, these observations suggest that reduction in the lipid mobility is not due to the metabolic glycan labeling nor the click conjugation of azide ligands and DBCO molecules, but rather due to the recruitment of DNA origami. Their large molecular weight and the potential spatial hindrance may be responsible for the low mobility of DNA origami. Our findings also show that the cross-linking of DNA rods further decreased the fluidity of membrane lipid, presumably due to enhanced rod–membrane interactions induced by rod cross-linking.

The cell membrane is vital for maintaining cell size, shape, and integrity, protecting the cell from outside assaults. The enhancement in membrane mechanics and the gelation of certain membrane lipids due to the nanoshell coating show the potential of the nanoshell in providing protection to cells under harsh and mechanically challenging environments. To investigate its protective potential, we first examined the cell viability after cells were coated with a nanoshell as a baseline. As expected, nanoshell-coated cells had high viability as the DNA nanostructures were biocompatible and the whole synthesis process was performed under physiological conditions (Supplementary Figure 7). We then applied osmotic imbalanced solutions to cells by changing the sodium chloride (NaCl) concentration from 0.9% to 0.6%, 0.3%, and 0%. The resulting cell sizes and viability were measured (Figure 5A–C). As the osmotic pressure decreased, we found a rapid increase in the sizes of native cells and a decrease in their viability, whereas nanoshell-coated cells maintained the cell size and had a ∼20% higher cell viability in lower osmotic solutions, with statistical differences compared to pretreatment native cells (Figure 5B,C). Notably, the nanoshell systems even maintained cell shape under 0% NaCl after a 10 min incubation. In contrast, native cells and rod A-coated cells burst rapidly within seconds. Although the rod A coating was also able to limit cell expansion, it was not able to rescue cell viability in low NaCl concentration solutions. We also noticed a slight decrease in the baseline cell size under 0.9% NaCl with 115 ± 30 μm^2^ for native cells, 109 ± 24 μm^2^ for rod A-coated cells, and 102 ± 20 μm^2^ for nanoshell-coated cells, with statistical difference between nanoshell-coated cells and rod A-coated cells. These findings demonstrate the utility of coating DNA origami on the membrane to maintain the cell size. Even a simple coating of rod A can limit cell expansion and improve survival, but the cross-linked nanoshell with both rods A and B is most effective in limiting expansion and importantly, acting as an armor and improving the survival of cells. Next, we applied centrifugal forces to cells and found that the nanoshell coating was able to rescue viability under 1500 and 3000 g (Figure 5D).^12^ Interestingly, the rod A coating was also able to improve cell viability against centrifugation, similar to its ability to limit expansion under osmotic swelling.

**Figure 5:**
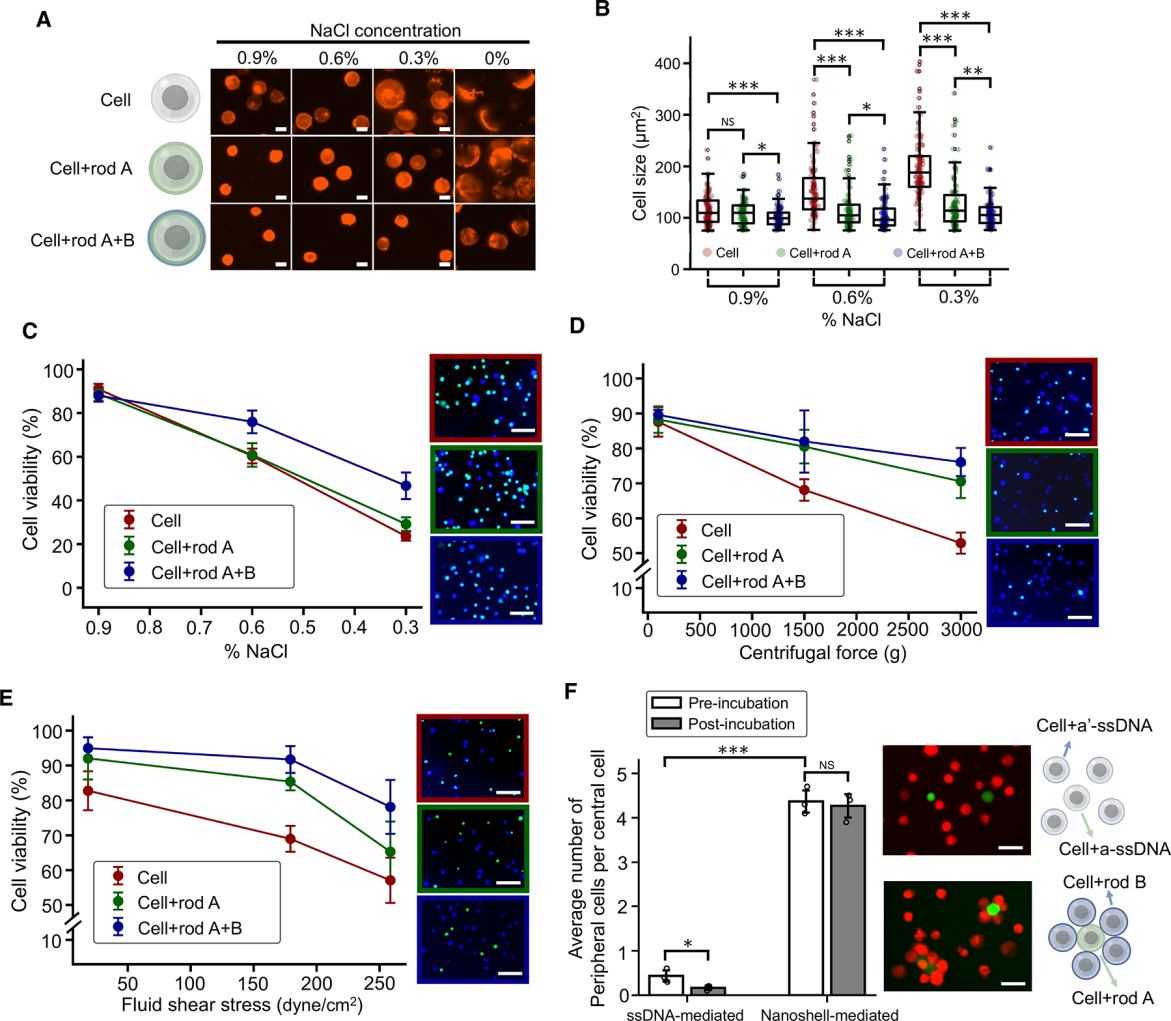
Protective effects of DNA nanoshell armor against challenging environments. (A) Fluorescence images of native cells, rod A-coated cells, and nanoshell-coated cells after 10 min incubation in a range of concentrations (mass per unit volume) of NaCl from 0.9% to 0.6% to 0.3% to 0%. Cell membranes were stained with DiD lipid dye (red). Representative images were shown for each group. Scale bars: 10 μm. (B) A bar plot for quantifying the cell size of native cells, rod A-coated cells, and nanoshell-coated cells under osmotic imbalanced solutions. *n* = 100. (C) The relationship between cell viability and incubation in osmotic imbalanced solutions. Three conditions of NaCl concentration, 0.9%, 0.6%, and 0.3%, were tested. Representative cell images of cells applied with 0.3% NaCl were shown. (D) The relationship between cell viability and centrifugation. A range of centrifugation rates were tested including 110*g*, 1500*g*, and 3000*g*. Representative cell images of cells applied with 3000 *g* were shown. (E) The relationship between cell viability and fluid shear stress in a syringe-pump model. A range of flow rates were tested, which resulted in the minimal fluid shear stresses of 18, 179, and 259 dyn/cm^2^. Representative cell images of cells applied with 259 dyn/cm^2^ were shown. Cells in (C)–(E) were live/dead stained. Blue: live. Green: dead. Scale bars for (C)–(E): 62.5 μm. (F) Multicellular assembly mediated by ssDNA and nanoshell. ssDNA-mediated assemblies were formed by a-ssDNA anchored cells (green) and a′-ssDNA anchored cells (red). Nanoshell-mediated assemblies were formed by rod A-coated cells (green) and rod B-coated cells (red). After cell assembly formation, all cells were incubated for 24 h and centrifugation was performed three times at 220 *g* for 5 min. Scale bars: 25 μm. Data were presented as means ± s.d., as indicated by error bars (*n* = 3). *P ≤ 0.05, **P ≤ 0.01, ***P ≤ 0.001.

Furthermore, using a syringe pump system to eject cells, we examined the cell viability under various fluid shear stresses.^42^ By changing the flow rate, 18, 179, and 259 dyn/cm^2^ of shear stress were applied to cells passing through the needle, according to Poiseuille’s equation,
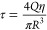
2where τ is the fluid shear stress. *Q* is the flow rate in cm^3^/s. η is the dynamic viscosity of the cell medium, which is treated as water (0.01 dyn s/cm^2^). *R* is the radius of the needle. The syringe ejection process was repeated 10 times. Our findings reveal that nanoshell-coated cells exhibit higher viability across all three shear stress conditions, suggesting that the nanoshell helps the cell resist against fluid shears (Figure 5E). Compared to native cells, nanoshell-coated cells showed a substantial increase in cell viability, rescuing up to 20% more cells. The mechanism of nanoshell formation through cross-linking can also be applied to facilitate multicellular assembly. Two groups of cells were immobilized with cell-surface anchors using the same concentration of cholesterol-a-ssDNA and cholesterol-a′-ssDNA, respectively. Subsequently, rod A and rod B (both having three a-ssDNA at the edge) were recruited onto the cell membranes. Comparing cell assembly mediated solely by ssDNA (using cells with only a/a′-ssDNA) and nanoshell-mediated assembly, where rod A and rod B cross-linked with each other to form assemblies, we found that the nanoshell-mediated assembly exhibited a significantly higher number of peripheral cells compared to the ssDNA-mediated assembly (Figure 5F). Furthermore, the effectiveness of the nanoshell-mediated assembly was maintained even after 24 h of incubation, followed by centrifugation, whereas the ssDNA-mediated assemblies were less robust. These results demonstrate several proof-of-concept applications of our DNA origami nanoshell, showcasing its ability to protect cells in challenging environments and its potential benefits for bioengineering applications such as cell printing and multicellular assembly.

In summary, we developed a modular strategy to encapsulate and ruggedize living cells using two layers of DNA origami nanorods. DNA rods were targeted and recruited to the cellular glycocalyx, followed by secondary rod cross-linking process, which successfully forms a biocompatible and biodegradable nanoshell under physiological conditions. We demonstrated that the composition of the nanoshell was tunable by modifying the valency and position of overhangs on rods. We investigated multiple properties of the nanoshell, including the improved stability and the evolving migration of rod distribution triggered by incubation and rod cross-linking, thereby showing the impact of dynamic interactions within DNA rod assemblies as well as between rods and cell membranes. By probing the membrane biomechanics of nanoshell-encapsulated cells, we found that the nanoshell increased the membrane elastic modulus and inhibited membrane lipid fluidity in a tunable manner. Importantly, the nanoshell provided extra protection to cells, supporting enhanced viability despite harsh osmotic imbalance, centrifugal forces, and fluid shear stress. Moreover, the cross-linking of DNA rods was shown to enable effective and robust multicellular assembly, providing new insight in manipulating cell–cell interactions.

As DNA origami have been increasingly applied to cell biology, our study sheds light on the interactions between DNA nanostructures and cell plasma membrane, including the stability of the membrane-bound DNA nanostructures and the biophysical influences on cell membranes.^22−25^ While the practical applications of DNA nanostructures have been hindered by cost and scalability issues, ongoing research efforts have focused on overcoming these challenges through advancements in manufacturing and structure design.^43,44^ As a result, the increasing utility and capability of systems such as our viability-enhancing nanoshell-armored cells demonstrate the potential benefit of this technology in biomedical and clinical applications such as cell printing and cell assembly.^10,12,17^

## Supporting Information Available

Full description of materials, methods and experimental details, including the design and sequences of DNA nanorods, synthesis of cell armor on live cells, examination of protective effects to cells, *ex vivo* syringe-needle ejection and nanoshell-mediated cell assembly, gel electrophoresis, nanorod and nanoshell coating, modulation of nanoshell composition, nanorods and nanoshell degradation, fluorescence recovery, cell viability, and lists of DNA oligos (PDF)
