# Supplementary material for: Synthetic Cell Armor Made of DNA Origami
Source: Nano Lett. 2023 Jul 18;23(15):7076–85. doi: 10.1021/acs.nanolett.3c01878 (PMC10416349; doi:10.1021/acs.nanolett.3c01878)
Supplement: Supplementary file 1 — nl3c01878_si_001.pdf [file nl3c01878_si_001.pdf]

Supporting Information

Synthetic cell armor made of DNA origami

Weitao Wang[1]*, Peter R. Hayes*[2]*, Xi Ren*[‡,1,3] *and Rebecca E. Taylor*[‡,1,3,4]

[1] *Department of Mechanical Engineering, Carnegie Mellon University, 5000 Forbes Avenue, Pittsburgh, Pennsylvania, United States.*

[2] *Department of Chemical Engineering, Carnegie Mellon University, 5000 Forbes Avenue, Pittsburgh, Pennsylvania, United States.*

[3] *Department of Biomedical Engineering, Carnegie Mellon University, 5000 Forbes Avenue, Pittsburgh, Pennsylvania, United States.*

[4] *Department of Electrical and Computer Engineering, Carnegie Mellon University, 5000 Forbes Avenue, Pittsburgh, Pennsylvania, United States.*

E-mail: xir@andrew.cmu.edu; bex@andrew.cmu.edu

List of Supplementary Information:

Supplementary Figure 1. Cadnano design of DNA nanorod.

Supplementary Figure 2. Gel electrophoresis studies of rod A formation with varying number of hybridization ssDNA (h-ssDNA).

Supplementary Figure 3. DNA nanorod and nanoshell coating of adherent HUVECs.

Supplementary Figure 4. Modulation of nanoshell composition by the concentrations of DNA rods.

Supplementary Figure 5. DNA nanorods and DNA nanoshell degradation by DNase I.

Supplementary Figure 6. Fluorescence recovery after photobleaching (FRAP) experiments of native cells, rod A-coated cells and nanoshell-coated cells.

Supplementary Figure 7. Cell viability of native cells in control buffers and cells after DNA rod and nanoshell coating.

Table S1. List of functional DNA oligos.

Table S2. List of DNA oligos for DNA nanorods.

Materials and Methods

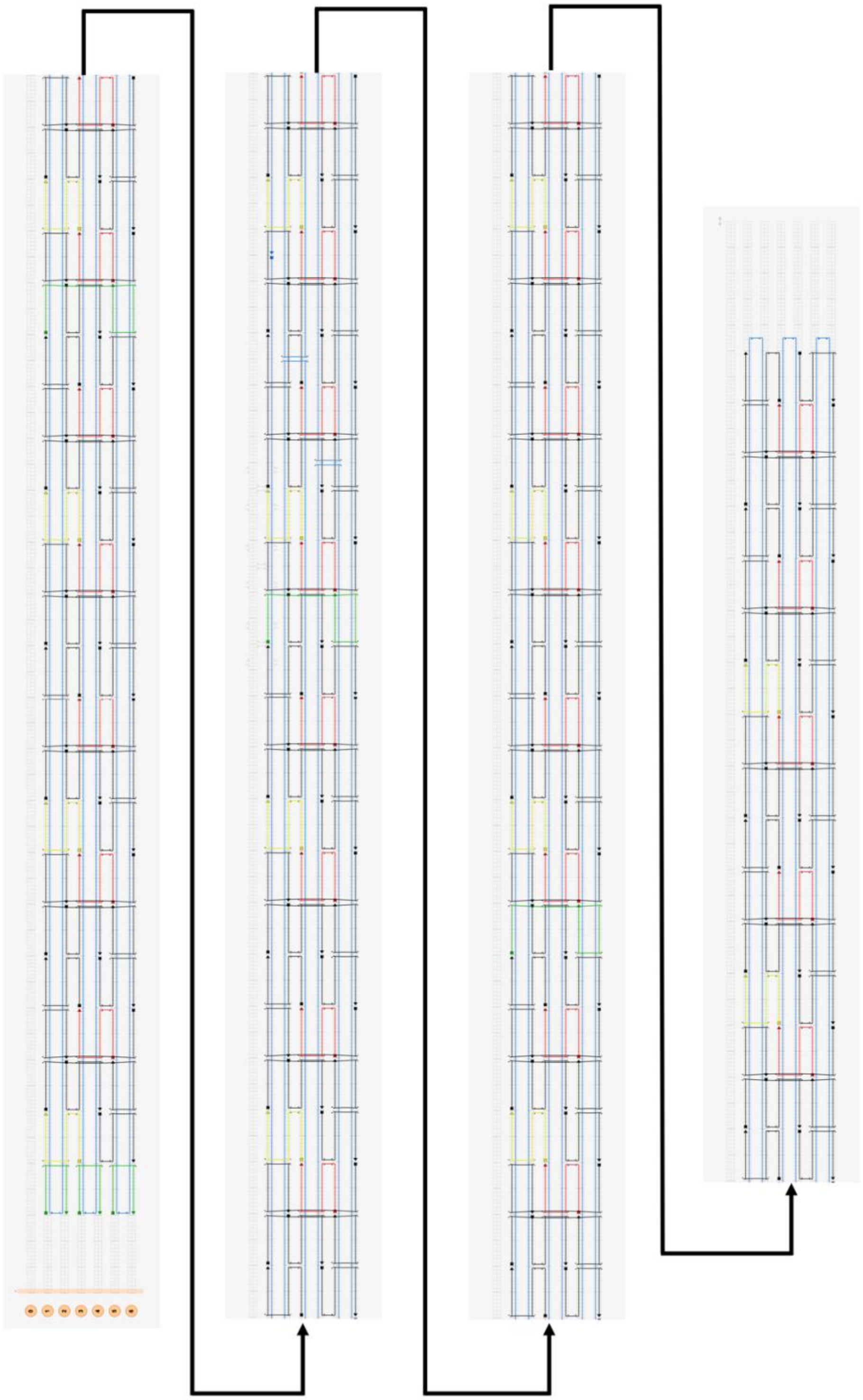

Supplementary Figure 1. Cadnano design of DNA nanorod. Green staples are modified with anchoring-ssDNA for rod A (rod B doesn't have green staples), 6 in total (3 at the edge are edge-decorated rod A. 3 in the center for center-decorated rod A). Yellow staples are modified with staining-ssDNA, 14 in total. Red staples are modified with hybridization-ssDNA for rod A and hybridization-ssDNA complementary for rod B, 28 potential sites in total. The naming of these sites is h-1 to h-28, from left to right.

Lane	1	2	3	4
a-ssDNA	3	3	3	3
s-ssDNA	14	14	14	14
h-ssDNA	7	14	21	28

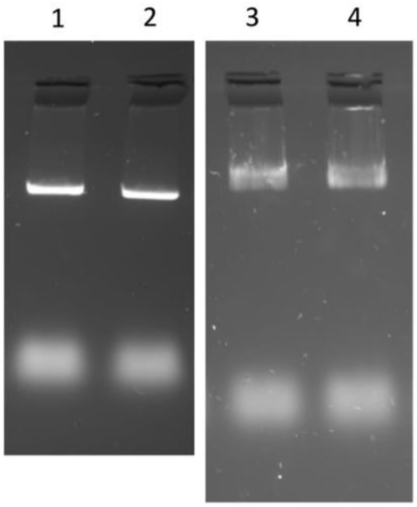

Supplementary Figure 2. Gel electrophoresis studies of rod A formation with varying number of hybridization ssDNA (h-ssDNA). Rod A were modified with 3 anchoring ssDNA, 14 staining ssDNA, and 7 or 14 or 21 or 28 h-ssDNA. Rod A had monodispersed individual bands when rod A was modified with 7 or 14 h-ssDNA, showing the formation of DNA origami rods. When modified with 21 or 28 h-ssDNA, rod A formation was not clear.

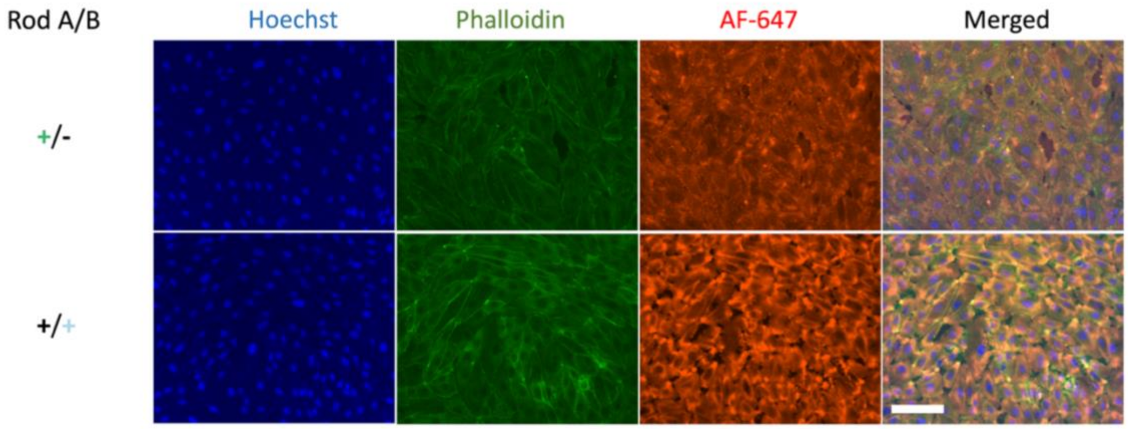

Supplementary Figure 3. DNA nanorod and nanoshell coating of adherent HUVECs. Cell nucleus were stained with Hoechst (blue). Cell membrane F-actin were stained with phalloidin (green). DNA nanorods were stained with AF-647. The experiment in the first row is cell + DNA rod A where rod A were stained. The experiment in the second row is cell + DNA rod A + rod B where rod B were stained. Scale bars: 125 μm.

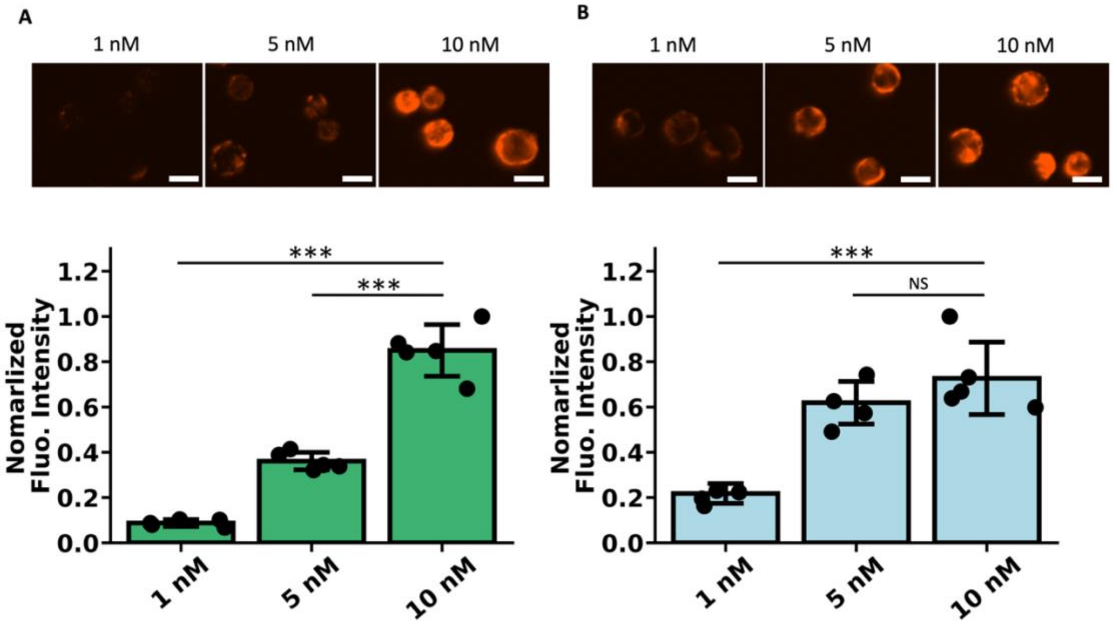

Supplementary Figure 4. Modulation of nanoshell composition by the concentrations of DNA rods. (A) The concentration of rod A was adjusted from 1 nM, 5 nM to 10 nM. The binding of rod A was measured by rod A fluorescence intensity (labeled with AF-647). (B) 5 nM of rod A were firstly administrated to cells. Then rod B were introduced where the concentration of rod B was adjusted from 1 nM, 5 nM to 10 nM. The binding of rod B was measured by rod B fluorescence intensity (labeled with AF-647). In both experiments, the volume of rods was 100 µl. Representative fluorescence microscopy images were taken. ***P ≤ 0.001 (n=5). All scale bars: 10 µm.

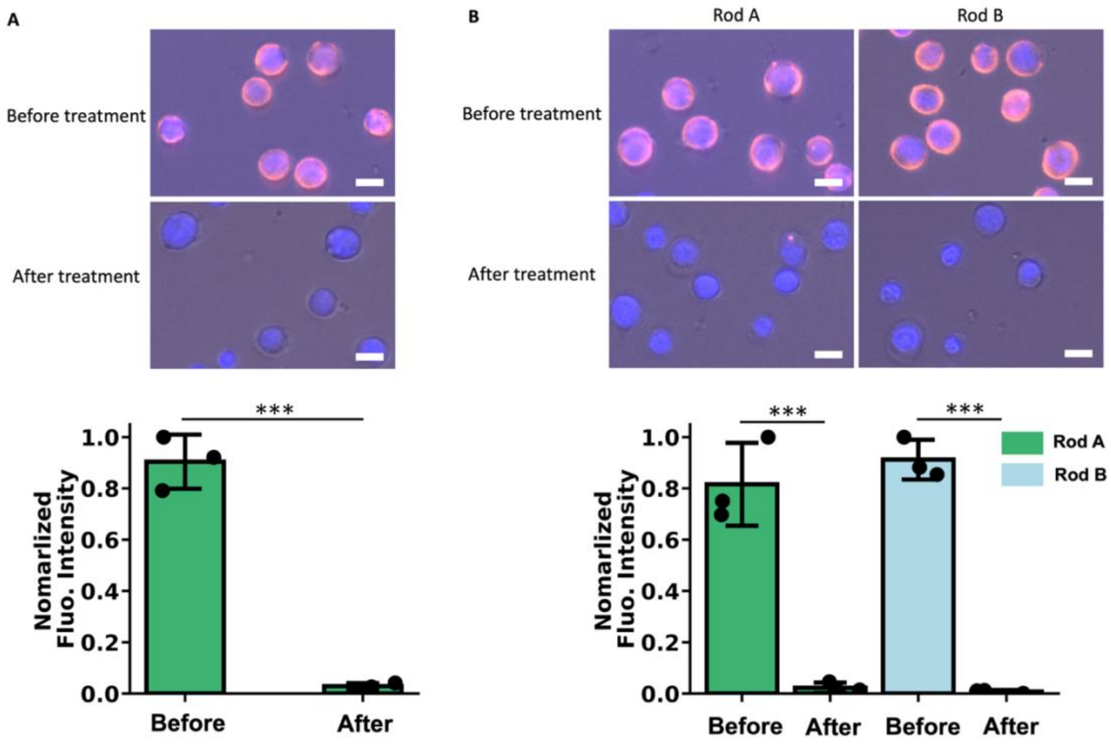

Supplementary Figure 5. DNA nanorods and DNA nanoshell degradation by DNase I. (A) Normalized fluorescence signal intensity of rod A on rod A-coated cells before and after DNase I treatment. (B) Normalized fluorescence signal intensity of rod A and rod B on nanoshell-coated cells before and after DNase I treatment. In both experiments, cell nucleus were stained with DAPI (blue). DNA rod A were stained with AF-647 (red). Representative bright-field and fluorescence images were shown. ***P ≤ 0.001. All scale bars: 10 μm.

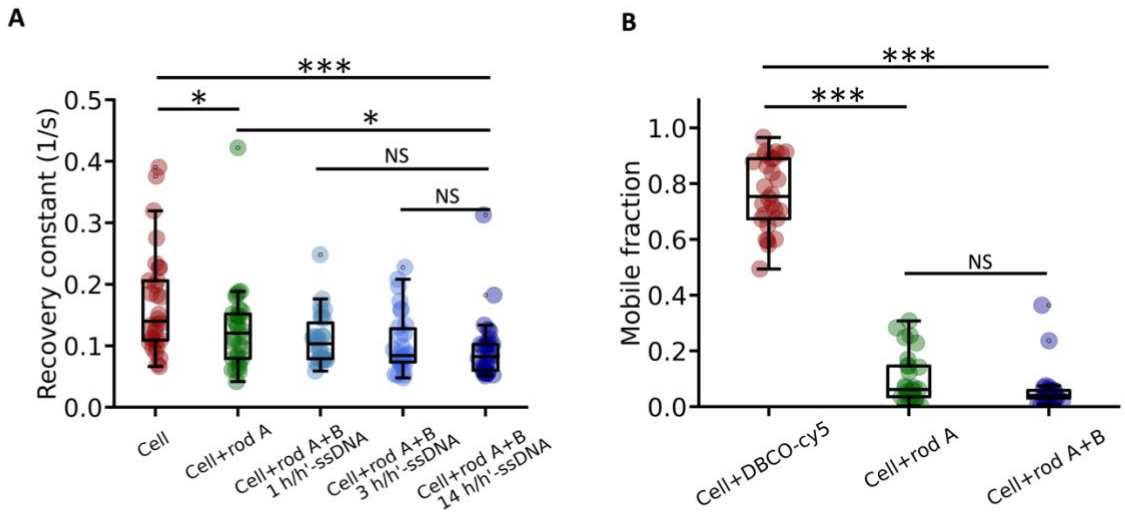

Supplementary Figure 6. Fluorescence recovery after photobleaching (FRAP) experiments of native cells, rod A-coated cells and nanoshell-coated cells. For nanoshell-coated cells, two conditions were included, where the rods were decorated with 1, 3 and 14 h/h'-ssDNA, respectively. (A) recovery constant of cell membrane lipids and (B) the mobility of DBCO-cy5 in native cell+DBCO-cy5, the mobility of rod A in rod A-coated cells and the mobility of rod A in nanoshell-coated cells. n=30. *P ≤ 0.05, ***P ≤ 0.001.

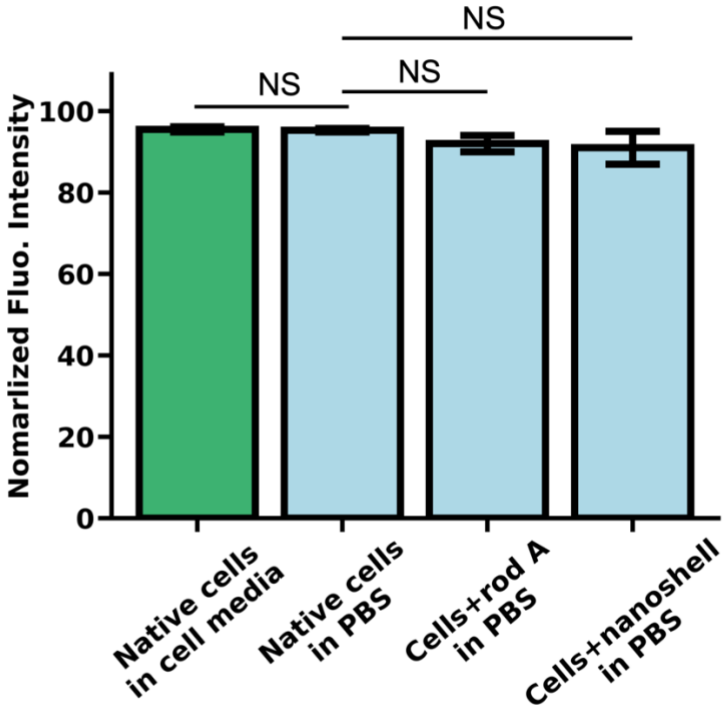

Supplementary Figure 7. Cell viability of native cells in control buffers and cells after DNA rod and nanoshell coating. Four conditions include native cells in cell media (RPMI+10%FBS), PBS and cells decorated with rod A and the nanoshell, both in PBS. Cell viability was assessed by ReadyProbes Cell viability imaging kit. Data are presented as mean ± standard deviation as indicated by error bars. n=3.

Materials and Methods

Materials, reagents and equipment

DNA oligos (Table. S1) were purchased from Integrated DNA Technologies (Coralville, IA). DBCO-cy5 and DBCO-Sulfo-NHS-Ester, Sodium chloride and paraformaldehyde and PEG 8000 were purchased from Sigma-Aldrich. Streptavidin-Alexa Fluor 488 and 647 conjugates were purchased from Invitrogen. Roswell park memorial institute (RPMI) 1640 medium, dulbecco's phosphate-buffered saline (DPBS) was purchased from Corning. Bovine serum albumin (BSA) was purchased from Fisher BioReagents. AFM tips were purchased form NanoAndMore. Micropipettes (5 μm in diameter) were purchased from World Precision Instruments. Membrane lipid dye DiD and DNase I were purchased from ThermoFisher. ReadyProbes Cell viability imaging kit (Blue/Green) and SYBR Safe DNA gel stain were purchased from Invitrogen. Endothelial cell growth medium-2 bulletKit was purchased from Lonza.

Synthesis and purification of biotinylated DNA nanorods

DNA nanorods were folded from M13mp18 scaffold (Bayou Biolabs) together with 173 staple strands (rod A) and 170 staples (rod B). For annealing, 20 nM of the scaffold and 100 nM of the staples were mixed in TAE buffer with 12.5 mM $MgCl_2$. The biotin-labeled strands were added pre-annealing with the same concentration as other staples. The mixture was heated to 80℃ for 5 min and gradually cooled down to 4℃ within 45 h. specifically, 80℃-65℃, 0.1℃ per 24 seconds; 65℃-24℃, 0.1℃ per 378 seconds; 24℃-4℃, 0.1℃ per 18 seconds. After annealing, the solutions were collected and precipitated by centrifugation at 10500 g for 25 min in a buffer containing 7.5% PEG 8000, 10 mM $MgCl_2$, 255 mM NaCl, 22.5 mM Tris, 10 mM acetic acid, and 1mM EDTA. The supernatant was removed and the pellet was resuspended to 5 nM using DPBS buffer with 12.5 mM $MgCl_2$. DNA nanorods were then administrated to cells within 30 min after purification.

Agarose gel electrophoresis and stability test

10 μl of 10 nM purified DNA nanorod samples were analyzed by electrophoresis in 2% agarose gel in TBE buffer with 12.5 mM $MgCl_2$ at 90 V and room temperature (RT) for 1 h. To study the structural stability of DNA nanorods in cell media, DNA rod A were incubated in EGM-2, RPMI+10% FBS at 37℃ for 6 h and 30 h before running the gel. To

test the stability of rod A and B aggregate stability, rod A and B were resuspended in RPMI+10% FBS at 20 nM and were mixed and incubated at 37℃ for 30 min and 30 h before running the gel. The gels were stained with 1×SYBR Safe DNA gel stain and imaged with Biorad ChemiDoc Imaging System.

Atomic force microscopy (AFM)

10 μl of 0.5 nM of purified DNA nanorod samples in DPBS with 12.5 mM $MgCl_2$ was deposited onto a freshly cleaved mica surface, incubated at RT in a humid chamber for 5 min. The sample was washed three times with 20 μl DI water and thoroughly blow-dried with nitrogen after each washing. AFM scans were performed using MFP-3D-BIO AFM (Asylum Research) with a 5 nM AFM tip in tapping mode.

Synthesis of 5'DBCO-a'-ssDNA

The 5'NH_2-a'-ssDNA oligos were incubated overnight in DPBS with DBCO-sulfo-n-hydroxysuccinimidyl ester (DBCO-Sulfo-NHS-Ester) at a 1:10 molar ratio under agitation at RT for 30 min. After incubation, the reaction mixture was dialyzed five times against DPBS using Amicon Ultra Centrifugal filters (molecular weight cut-off, 3 kDa) to remove unconjugated DBCO-Sulfo-NHS-Ester. The above-described conjugation and purification procedures were repeated twice in total to increase the conjugation efficiency. The final concentration of 5'DBCO-a'-ssDNA was adjusted to 500 μM with DPBS.

Cell culture

Jurkat, clone E6-1, were purchased from ATCC. Cells were cultured in RPMI+10% FBS at 37℃ with 5% CO_2 and were maintained at a cell density between 100,000 cells/ml and 3,000,000 cells/ml. Human umbilical vein endothelial cells (HUVECs) were purchased from the Lonza. Cells were cultured in EGM-2 at 37℃ with 5% CO_2. HUVECs of passage 3-5 were plated into 96-well culture plates at a density of 50,000 cells per well, one day prior to the experiment. Both cells have been tested negative for mycoplasma contamination.

Synthesis of DNA nanoshell on membrane through click chemistry and ssDNA hybridization

In the first step, we immobilized a'-ssDNA on glycocalyx. Azide ligands were firstly covalently incorporated onto glycocalyx 24 h before experiment, through metabolic glycan labeling using an azido monosaccharide, N-azidoacetylmannosamine-tetraacylated (Ac4-ManNAz). Specifically, Ac4ManNAz was diluted in culture medium to a final concentration of 50 μM and administered to cells 24 h before experiment. DBCO-a'-ssDNA was administrated to Jurkat cells at a final concentration of 50 μM and 100 μl in volume and incubated for 1 h. Cells were then washed with DPBS+0.1% BSA as washing buffer to remove excess DBCO-a'-ssDNA using centrifugation at 200 g for 3 min. Next, 100 μl of 5 nM DNA rod A in DPBS with 12.5 mM $MgCl_2$ were administrated to cells and incubated for 30 min. Then 100 μl of 5 nM DNA rod B in DPBS with 12.5 mM $MgCl_2$ were administrated to cells and incubated for 30 min. Cells then were washed and resuspended in DPBS or RPMI+10% FBS buffers for later experiments.

Examination of the cell surface retention time of the nanoshell with flow cytometry

In this experiment, only DNA rod A were biotinylated and fluorescently labeled. Cells decorated with only rod A and with both rods A and B were incubated at three conditions: 4℃ for 30 min, (ii) 37℃ for 30 min and (iii) 37℃ for 3 h, with RPMI1640+10% FBS as buffer. After incubation, cells were collected and fixed using 1 ml of 4% of paraformaldehyde with 10 min at RT. DPBS was used for washing. DNA rod A were then stained with streptavidin-AF647 and cells were washed. Finally, cells were analyzed using flow cytometry and fluorescence intensity of AF647 was examined by FACS.

Degradation of DNA nanorods and nanoshells on cell membranes

DNA rod A-coated cells and nanoshell-coated cells (1,000,000 cells/ml) were incubated at 37℃ for 30 min with a concentration of DNase I at 20 U and with DPBS as buffer. After incubation, cells were washed and imaged.

Examination of nanoshell internalization

Two groups of cells, rod A-coated cells and nanoshell-coated cells were prepared and incubated at 37℃ for 30 min. In both groups, only rod A were fluorescently labeled with AF647. After incubation, cells were fixed using 4% paraformaldehyde. DNase I at 20 U was introduced to remove cell-surface DNA rods. The background fluorescence noises

were removed and the internalization signal intensity were quantified using a previously reported computational pipline.[1]

Quantification of fluorescence signal distribution on cell border

The fluorescence signals on cell borders were acquired using National Institutes of Health Image J by manually circling cell borders and recording the intensity.[2] The data were then analyzed by a custom Python 3.8 code using SciPy where the signal valley was defined as a fluorescence signal that was lower than 0.4, and the number and length of signal valleys were quantified.[3]

Examination of membrane lipid fluidity with FRAP

Live native cells, rod A-coated cells and nanoshell-coated cells were observed under confocal laser scanning microscope (LSM 880 with Airyscan, ZEISS). Cell membranes were stained with DiD lipid dye (red) and DNA rod A were stained with AF488 (green). Region of interest (ROI) was selected and set to be photobleached. The pinhole size for photobleaching was 2 μm. After photobleaching, the relation of fluorescence recovery with time was recorded and analyzed to calculate the mobile fraction, $t_{0.5}$ and recovery constant by ZEN (ZEISS).

Examination of cell membrane elastic modulus with micropipette aspiration

Live native cells, rod A-coated cells and nanoshell-coated cells were deformed by glass micropipettes. Specifically, the micropipette was connected to tubing that ran to a water-filled reservoir with controllable height. The tip of the micropipette was then placed near a cell and a suction pressure was applied within the micropipette. The pressure difference deformed the cell and a membrane protrusion was formed. The length of the protrusion, the aspirated length, was recorded. Pressure difference and aspirated length were fitted linearly by forcing the fitted line to pass through the origin. Elastic modulus was calculated using the model and equation by Theret *et al*., assuming the cell as a continuum-medium model with homogeneity.[4]

Protective effect of the nanoshell against osmotic swelling and centrifugal forces

In the osmotic swelling assay, live native cells, rod A-coated cells and nanoshell-coated cells were diluted to a concentration of 1,000,000 cells/ml. Cell membranes were stained

by DID lipid dye (red). Cells were then centrifuged and resuspended in 1 ml of NaCl solutions (0%, 0.3%, 0.6%, 0.9%). After 10 min incubation at 37℃, cells were collected and imaged by a fluorescence microscope (EVOS). The sizes of cells were analyzed using Image J software by applying the same threshold to red areas in all images. To test cell viability, when incubating cells, NaCl buffers were mixed with cell viability imaging kit solutions. After incubation, cells were imaged on EVOS at 40x in both Cy5 and GFP channels. In the centrifugal assay, cells (1,000,000/ml) were centrifuged (centrifuge 5804R, eppendorf) five times at 4℃ with a speed of 100 g, 1500 g and 3000 g, respectively. After each centrifugation, cells were resuspended by 1 ml of DPBS. Cells were then incubated in cell viability imaging kit solutions at 37℃ for 10 min, followed by fluorescence microscopy imaging. Dead cells were stained green. All cells were stained blue. Cell viability was calculated as the ratio of live cell/all cells.

Protective effect of the nanoshell against *ex vivo* syringe-needle ejection-induced fluid shear stress

Live native cells, rod A-coated cells and nanoshell-coated cells were collected at 500,000 cells/ml in RPMI+10% FBS cell medium. 5 ml of cell suspension was slowly loaded into a 20 ml syringe (BD Biosciences). A 30 G needle (Jensen Global) was then attached to the syringe. An automated syringe pump (New Era NE-1000) was prepared and the pumping rate was adjusted to 0.6, 5.8 and 8.4 ml/min, respectively, corresponding to 10, 97 and 140 μl/s flow rate. At room temperature, under each pumping rate, cells were ejected through the needle and suspension was collected. The process was repeated 10 times. Cell viability was examined using ReadyProbes Cell viability imaging kit.

DNA nanoshell-mediated cell assembly

First, two groups of Jurkat cells were fluorescently stained with CellTracker Green CMFDA Dye and CellTracker Deep Red Dye, respectively. One group of cells were incubated with cholesterol-a-ssDNA and the other group of cells were incubated with cholesterol-a'-ssDNA. Both incubations were 1 h long. For ssDNA-mediated cell assembly, two group of cells (cell surface immobilized with cholesterol-a-ssDNA and cholesterol-a'-ssDNA) were

mixed at a ratio of 1:100 (10,000 cells/ml : 1,000,000 cells/ml) at 37℃ for 30 min. For DNA nanoshell-mediated cell assembly, two groups of cells were firstly incubated with rod A (3 a-ssDNA, 14 h-ssDNA) and rod B (3 a-ssDNA, 14 h'-ssDNA, where the positions of a-ssDNA are the same of rod A) at 37℃ for 30 min, respectively, followed by centrifugation washing. Then two groups of cells were mixed at 1:100 ratio for another 30 min incubation at 37℃. Following assembly, cells were transfer to a 96 well plate for fluorescence imaging using the EVOS system. To further test the robustness of the cell assembly, assembled cells were incubated 24 h, followed by three times of centrifugation at 200g for 5 min. Cell assembly were then assessed again by EVOS system.

Statistics and Reproducibility

Quantitative data were displayed as means ± s.d. Statistical significances were determined using one-way analysis of variance (ANOVA) with post-hoc Tukey's Test. All cell experiments were repeated independently at least three times.

Table S1. List of functional DNA oligos.

Name of oligos	Sequence
Anchoring-ssDNA (a-ssDNA) modifier	5'/TT CAGTCAGTCAGTCAGTCAGT/3'
staining-ssDNA (s-ssDNA) modifier	5'/TT GAGAGCAGACCTGGAACTCG/3'
hybridization-ssDNA modifier (h-ssDNA, for rod A)	5'/TT GTGACATACCTTCGGAGCAT/3'
hybridization-ssDNA complementary modifier (h'-ssDNA, for rod B)	5'/TT ATGCTCCGAAGGTATGTCAC/3'
5'NH_2-a'-ssDNA	/5AmMC6/TT ACTGACTGACTGACTGACTG
Biotin-s'-ssDNA	/5BiosG/TT CGAGTTCCAGGTCTGCTCTC
Cy5-s'-ssDNA modifier	/5Cy5/TT CGAGTTCCAGGTCTGCTCTC
Cy3-s'-ssDNA modifier	/5Cy3/TT CGAGTTCCAGGTCTGCTCTC
Chol-a'-ssDNA	5'/Chol-TEG/ACTGACTGACTGACTGACTG/3'
Chol-a-ssDNA	5'/Chol-TEG/CAGTCAGTCAGTCAGTCAGT/3'

Table S2. List of DNA oligos for DNA nanorods.

color in cadnano file	sequence	+a-ssDNA	+s-ssDNA	+1 h/h'-ssDNA	+3 h/h'-ssDNA	+7 h/h'-ssDNA	+14 h/h'-ssDNA
black	TCAGGCTGCGCAACCTAGGGCGCTGGCAATCGTCTGAAATGG						
black	CATAACGCCAAAAGTTGCTAAACAACTTCCAATAGGAACCCA						
black	CCGCTTCTGGTGCCCCACACCCGCCGCGACAGGAAAAACGCT						
black	TATCGGCCTCAGGAATGGTTGCTTTGACTTGCTGGTAATATC						
black	CATCGTAACCGTGCGAATCAGAGCGGGAATAACATCACTTGC						
black	GATTGACCGTAATGTTAGACAGGAACGGTCACGCAAATTAAC						
black	TCAGTTGAGATTTAAAGGAACAACTAAACCACCCTCAGAGCC						
black	AACGAACTAACGGATGAAAATCTCCAAAGGTTTAGTACCGCC						
black	TATACCAGTCAGGAGTATCGGTTTATCAATATAAGTATAGCC						
black	ATCATTGTGAATTAAGCTTGATACCGATTTTTGCTCAGTACC						
black	CGAGTAGTAAATTGGCCCACGCATAACCAGAGGCTGAGACTC						
black	TCATTCAGTGAATAGAGTTAAAGGCCGCTGCCTATTTCGGAA						
black	AGAACCGGATATTCAAAGACAGCATCGGGTGCCTTGAGTAAC						
black	GGCGCATAGGCTGGTTGAGGACTAAAGAGATGATACAGGAGT						
black	TGACCAACTTTGAAGGGTAAAATACGTATCTCTGAATTTACC						
black	GCCGGAACGAGGCGCGAAAGAGGCAAAACAAACAAATAAATC						
black	GATAAATTGTGTCGCCCAGCGATTATACGAAGTATGTTGAGG						
black	TTGCGTATTGGGCGCTTTTCACCAGTGAAATAGATTAGAGCC						
black	TATCATAACCCTCGCGTCTTTCCAGACGGTACAAACTACAAC						
black	CAGCTGCATTAATGGCCTGGCCCTGAGATGAGGAAGGTTATC						
black	TTGCGCTCACTGCCTGCCCCAGCAGGCGATCAATATCTGGTC						
black	GCCTGGGGTGCCTATCGGCAAAATCCCTTCTAAAGCATCACC						
black	ACAATTCCACACAAGGTTGAGTGTTGTTCCTGCAACAGTGCC						
black	TCATGGTCATAGCTAGAACGTGGACTCCGCAGAAGATAAAAC						
black	TCGACTCTAGAGGAAGGGCGATGGCCCAAGCCCTAAAACATC						
black	GTTGTAAAACGACGTTTTGGGGTCGAGGAATATTTTTGAATG						
black	GGCGATTAAGTTGGAAGGGAGCCCCCGAGAACCCTTCTGACC						
black	CTTCGCTATTACGCACGTGGCGAGAAAGACACGACCAGTAAT						
black	GAGAAGTGTTTTTAGTCGGATTCTCCGTAAATGTGAGCGAGT						
black	ATTATTTACATTGGAATTAATTACATTTCGTTATACAAATTC						
black	CATGGAAATACCTAATGGAAACAGTACACGGAATCATAATTA						
black	CAGAACAATATTACTGCTTCTGTAAATCACCGACCGTGTGAT						
black	CTGAGTAGAAGAACTCCTTGAAAACATATAGTTAATTTCATC						
black	CGTTGTAGCAATACGAGTCAATAGTGAATCGCAAGACAAAGA						
black	GAGGCCACCGAGTAACCTTTTTAACCTCGTTGGGTTATATAA						
black	ACCACCCTCATTTTCAAAGACAAAAGGGAACAAAGTTACCAG						
black	ACCCTCAGAACCGCGTAAATATTGACGGATCTTACCGAAGCC						
black	CGGAATAGGTGTATCGTCACCGACTTGAAGCCCAATAATAAG						
black	AGGCGGATAAGTGCCACCAGTAGCACCATGAGCGCTAATATC						
black	CTCAAGAGAAGGATCAATGAAACCATCGGGGAGAATTAACTG						
black	CCTATTATTCTGAAAATCAAGTTTGCCTTTTACAGAGAGAAT						
black	AGTGCCCGTATAAACGGCATTTTCGGTCGAAACGATTTTTTG						
black	GTACTGGTAATAAGTTTCATAATCAAAATTACAAAATAAACA						
black	GTTCCAGTAAGCGTCGCCTCCCTCAGAGTACCAACGCTAACG						
black	GCCTGTAGCATTCCCAACATATAAAAGAGCAGTATGTTAGCA						
black	CTCATTAAAGCCAGAGCCACCACCCTCATAGTTGCTATTTTG						
black	CAGGTCAGACGATTCGCCGCCAGCATTGACCTCCCGACTTGC						
black	GTCAATAGATAATACAACTCGTATTAAAAAGGCTTATCCGGT						
black	TAAAATATCTTTAGAAGTTTGAGTAACAAGGAATCATTACCG						
black	AGTTGGCAAATCAACAGAAGGAGCGGAACGCACTCATCGAGA						
black	TTGCTGAACCTCAAATGGCAATTCATCAGTCTTTCCTTATCA						
black	ACGCTGAGAGCCAGTCTGAATAATGGAAATCCTAATTTACGA						
black	AGAGGTGAGGCGGTTTGCACGTAAAACATATCAACAATAGAT						
black	GCCATTAAAAATACGTTTAACGTCAGATGACAATAAACAACA						
black	GCTATTAGTCTTTACGGGAGAAACAATAATAAAGTACCGACA						
black	TGTACCGTAACACTTTTTGTCACAATCAGGAATACCCAAAAG						
black	TGAAAGCGTAAGAAAAGTTACAAAATCGGTAATTTAGGCAGA						
black	AAAAGGGACATTCTCCTGAGCAAAAGAATAGGGCTTAATTGA						
black	TTACCAGTATAAAGCGGTAATCGTAAAAATCGGTGCGGGCCT						
black	CTAGAAAAAGCCTGTGATAATCAGAAAAGCGCCATTCGCCAT						
black	AAATAAGGCGTTAAAAATATTTAAATTGCCAGCTTTCCGGCA						
black	TTCTGACCTAAATTATTAAATTTTTGTTGGGGACGACGACAG						
black	ACGCGAGAAAACTTACGCCATCAAAAATGGTGTAGATGGGCG						
black	AAGGAAACCGAGGACGTCATAAATATTCAACTAATGCAGATA						
black	CTATATGTAAATGCCTTTCATCAACATTGGGAACAAACGGCG						
black	CTTTTTAAGAAAAGGTTCAGAAAACGAGGGTAGAAAGATTCA						
black	AGCAAGAAACAATGTACCCTGACTATTAATCTACGTTAATAA						
black	AGAGAGATAACCCAAAAGATTAAGAGGAAAGAACTGGCTCAT						
black	AACACCCTGAACAATAATTCGAGCTTCATAATTTCAACTTTA						
black	AACATAAAAACAGGACAGGTCAGGATTAGAGAAACACCAGAA						
black	TTTAACGTCAAAAAAAGAGGTCATTTTTCGTAACAAAGCTGC						
black	GCCATATTATTTATTATAATGCTGTAGCGAGTAATCTTGACA						
black	AGCGTCTTTCCAGATACGGTGTCTGGAACGGTGTACAGACCA						
black	CACCCAGCTACAATAATTCTGCGAACGATAAGGGAACCGAAC						
black	GGGAGGTTTTGAAGTTCGCAAATGGTCACTCCATGTTACTTA						
black	ATTCTAAGAACGCGGGGCGCGAGCTGAATTGTATCATCGCCT						
black	CGCCCAATAGCAAGTAGCATTACATCCACGGGGAGAGGCGGT						
black	ACAAGCAAGCCGTTAGCAAAATTAAGCAGAAACCTGTCGTGC						
black	TTCCAAGAACGGGTGGTTGTACCAAAAATCACATTAATTGCG						
black	GCATGTAGAAACCAAGAAGCCTTTATTTGCATAAAGTGTAAA						
black	AACTGGCATGATTATAGTAAAATGTTTAAGTAAGAGCAACAC						
black	AAGTCCTGAACAAGCTCATATATTTTAAATTGTTATCCGCTC						
black	TGTTCAGCTAATGCAGATTCAAAAGGGTGCTCGAATTCGTAA						
black	AAAGGTAAAGTAATATCAATATGATATTTGCATGCCTGCAGG						
black	GGCATTTTCGAGCCGGAGAGGGTAGCTATTCCCAGTCACGAC						
black	GAATCGCCATATTTGTCATTGCCTGAGAGGGATGTGCTGCAA						

color in cadnano file	sequence	+a-ssDNA	+s-ssDNA	+1 h/h'-ssDNA	+3 h/h'-ssDNA	+7 h/h'-ssDNA	+14 h/h'-ssDNA
black	CTGAGGCTTGCAGGAGGCTTGCCCTGACGAGAGTACCTTTAA						
black	AAATCGGAACCCTAGTAACGCCAGGGTTTTTTTGAGAGATCT						
black	ATATGTACCCCGGTTTTAGTATCATATGAACAATTTCATTTG						
black	AAGATTGTATAAGCATAAGAATAAACACTAAATCAATATATG						
black	TTGTTAAAATTCGCTAATGGTTTGAAATGTCGCTATTAATTA						
black	TTTAACCAATAGGATTTCAAATATATTTGCGATAGCTTAGAT						
black	CTTCCTGTAGCCAGTGATGCAAATCCAATTTATCAAAATCAT						
black	AAATGCTTTAAACATAAGCAGATAGCCGCGACATTCAACCGA						
black	AAAAATCAGGTCTTAAATAGCAATAGCTAAATTATTCATTAA						
black	GCGGATTGCATCAACAAGAATTGAGTTAGCCATTTGGGAATT						
black	GGAAGCAAACTCCAGAAGCGCATTAGACATAGCAGCACCGTA						
black	TTGCTCCTTTTGATTGAAAATAGCAGCCTTAGCGTCAGACTG						
black	GCTTAATTGCTGAACCCAATCCAAATAAATAGCCCCCTTATT						
black	ATATGCAACTAAAGGCCTAATTTGCCAGTCACCGGAACCAGA						
black	AACAGTTGATTCCCTTTATCCTGAATCTCCGCCACCCTCAGA						
black	GCCAGAGGGGGTAAAGACTCCTTATTACAACGCAAAGACACC						
black	TATATTTTCATTTGAGGCGTTTTAGCGAACAGGAGTAGACTT						
black	TCTACTAATAGTAGCAAATCAGATATAGTCCTTTGCCCGAAC						
black	CAAGGCAAAGAATTTTTATTTTCATCGTTTATCATTTTGCGG						
black	CATAAAGCTAAATCATTAAACCAAGTACTTATCATCATATTC						
black	AATACTTTTGCGGGATCAATAATCGGCTATATAATCCTGATT						
black	TAATGTGTAGGTAAAGAACGCGCCTGTTGAAATAAAGAAATT						
black	ACAGTCAAATCACCTCTGTCCAGACGACGAATATACAGTAAC						
black	GATAAATTAATGCCAGTAATAAGAGAATACGGATTCGCCTGA						
black	CAATACTGCGGAATAACGCAATAATAACATAGAAAATTCATA						
black	ACAAAGGCTATCAGAACAACGCCAACATCGCAGAGGCGAATT						
black	AGAGAATCGATGAACCAACGCTCAACAGGATGATGAAACAAA						
black	GAAAGGAGCGGGCGTGTTGGGAAGGGCGCTAGCATGTCAATC						
black	ACAGGGCGCGTACTAGATCGCACTCCAGTAAACGTTAATATT						
black	CGATTAAAGGGATTGGATAGGTCACGTTAATTCGCGTCTGGC						
black	TAATTTTTTCACGTACAACATTATTACAAATGACCATAAATC						
black	TGAATTTCTTAAACCCTTATGCGATTTTAGCCCGAAAGACTT						
black	CGGCTACAGAGGCTCTGACCTTCATCAATCAACATGTTTTAA						
black	GCACCAACCTAAAACAGACGGTCAATCAGTAGATTTAGTTTG						
black	AGTACAAGGTTTTTCCAGGGTCGGAGATAAGGTGGCATCAAT						
black	GGTCCACGCTGGTTCGCTTTCCAGTCGGATAAAGCCTCAGAG						
black	ATAGCCCGAGATAGCATACGAGCCGGAACAACGCAAGGATAA						
black	TTCTGTATGGGATTGAATTACGAGGCATGACTGGATAGCGTC						
black	AAAAACCGTCTATCTCCCCGGGTACCGAGAGAAAGGCCGGAG						
green	AGCGAGAGGCTTTTATAAAAACCAAAAT	yes (edge)					
green	TAGTTAGCGTAACGACAGACAGCCCTCA	yes (edge)					
green	ATACATAAAGGTGGAACGTAGAAAATAC	yes (edge)					
green	CAAATATCGCGTTTAGTCAGAGGGTAATTTACCATTAGCAAG	yes (center)					
green	ACCATTAGATACATCCTTAAATCAAGATGAGCCGCCACCAGA	yes (center)					
green	AAATTTTTAGAACCAAAAATAATATCCCGGGTTAGAACCTAC	yes (center)					
yellow	GCTGCGCGTAACCAGGAAACCAGGCAAAGCCCCAAAAACAGG		yes				
yellow	TGCTTTCCTCGTTAATCTGCCAGTTTGAAAATCAGCTCATTT		yes				
yellow	GGAGTGAGAATAGAGGAATACCACATTCATTGAATCCCCCTC		yes				
yellow	AGGAGCCTTTAATTCGTTGGGAAGAAAATAGTCAGAAGCAAA		yes				
yellow	TGACAACAACCATCGGCTTGAGATGGTTAAGCGAACCAGACC		yes				
yellow	ATCTAAAGTTTTGTTTTACCAGACGACGGCAAAAGAAGTTTT		yes				
yellow	CACCCTCAGCAGCGATTACCCAAATCAAGCGGATGGCTTAGA		yes				
yellow	AGTTTCCATTAAACAGAGGACAGATGAAGTTTCATTCCATAT		yes				
yellow	ACTCATCTTTGACCAAATCCGCGACCTGATAACCTGTTTAGC		yes				
yellow	GATTGCCCTTCACCAATCGGCCAACGCGATAAATCATACAGG		yes				
yellow	TGGTGGTTCCGAAAATGAGTGAGCTAACCATTATGACCCTGT		yes				
yellow	GAGTCCACTATTAAGTTTCCTGTGTGAAATGCAATGCCTGAG		yes				
yellow	ACCCAAATCAAGTTGCCAGTGCCAAGCTCAACCGTTCTAGCT		yes				
yellow	GGGAAAGCCGGCGACAGCTGGCGAAAGGGTCTGGAGCAAACA		yes				
red	ACGGAATAAGTTTAGAGTTTCGTCACCATTAGTAAATGAATT				yes	yes	yes
red	TGGTTTACCAGCGCCAGGGATAGCAAGCTCAACAGTTTCAGC						
red	TTGAGGGAGGGAAGCACCCTCAGAACCGGGAATTGCGAATAA						yes
red	AGGTGAATTATCACCACCGTACTCAGGAAAAAAGGCTCCAAA						
red	AGAGCCAGCAAAATCGTCGAGAGGGTTGGCTTGCTTTCGAGG					yes	yes
red	GCCGGAAACGTCACTAGGATTAGCGGGGAGTTGCGCCGACAA						
red	ATCAGTAGCGACAGACATGAAAGTATTAGATATATTCGGTCG						yes
red	TAGCGCGTTTTCATCAGTTAATGCCCCCTTTTGCGGGATCGT						
red	AGCGTTTGCCATCTTTTTAACGGGGTCAAACGAGGGTAGCAA					yes	yes
red	GCCACCACCGGAACCATACATGGCTTTTCTTTTTCATGAGGA						
red	ACCGCCACCCTCAGAATGGAAAGCGCAGATGCCACTACGAAG						yes
red	ACCACCACCAGAGCGGCCTTGATATTCAGAATACACTAAAAC						
red	TACAAACAATTCGACATTTGAGGATTTACAAGCGCGAAACAA			yes	yes	yes	yes
red	GTTATTAATTTTAAGAGCACTAACAACTGACGGGCAACAGCT						
red	AACAAAGAAACCACCAGTTGAAAGGAATGAGTTGCAGCAAGC						yes
red	CTGATTATCAGATGATATCAAACCCTCAAAAATCCTGTTTGA						
red	GTTTGGATTATACTCAGCAAATGAAAAATATAAATCAAAAGA					yes	yes
red	CATATCAAAATTATCAGTATTAACACCGCCAGTTTGGAACAA						
red	GCGTAGATTTTCAGCGAACGAACCACCAAACGTCAAAGGGCG						yes
red	AGTACCTTTTACATATGCGCGAACTGATCTACGTGAACCATC						
red	TTGCTTTGAATACCTACGTGGCACAGACTGCCGTAAAGCACT					yes	yes
red	ATTCATTTCAATTAGGCCAACAGAGATATTTAGAGCTTGACG						
red	CATCAAGAAAACAACAGATTCACCAGTCGAAGGGAAGAAAGC						yes
red	AATTACCTTTTTTACATTTTGACGCTCAAGTGTAGCGGTCAC						
red	TGAGTGAATAACCTCGCCAGCCATTGCACTTAATGCGCCGCT				yes	yes	yes
red	ATTTTCCCTTAGAATCAAACTATCGGCCGAGCACGTATAACG						
red	TAAGACGCTGAGAATTCTTTGATTAGTAGCTAAACAGGAGGC						yes
red	AGGTCTGAGAGACTAAAGAGTCTGTCCATACGCCAGAATCCT						

References

(1) Wijesekara, P.; Liu, Y.; Wang, W.; Johnston, E. K.; Sullivan, M. L. G.; Taylor, R. E.; Ren, X. Accessing and Assessing the Cell-Surface Glycocalyx Using DNA Origami. *Nano Lett.* **2021**, *21* (11), 4765–4773. https://doi.org/10.1021/acs.nanolett.1c01236.

(2) Schneider, C. A.; Rasband, W. S.; Eliceiri, K. W. NIH Image to ImageJ: 25 Years of Image Analysis. *Nat. Methods* **2012**, *9* (7), 671–675. https://doi.org/10.1038/nmeth.2089.

(3) Virtanen, P.; Gommers, R.; Oliphant, T. E.; Haberland, M.; Reddy, T.; Cournapeau, D.; Burovski, E.; Peterson, P.; Weckesser, W.; Bright, J.; van der Walt, S. J.; Brett, M.; Wilson, J.; Millman, K. J.; Mayorov, N.; Nelson, A. R. J.; Jones, E.; Kern, R.; Larson, E.; Carey, C. J.; Polat, İ.; Feng, Y.; Moore, E. W.; VanderPlas, J.; Laxalde, D.; Perktold, J.; Cimrman, R.; Henriksen, I.; Quintero, E. A.; Harris, C. R.; Archibald, A. M.; Ribeiro, A. H.; Pedregosa, F.; van Mulbregt, P.; SciPy 1.0 Contributors; Vijaykumar, A.; Bardelli, A. P.; Rothberg, A.; Hilboll, A.; Kloeckner, A.; Scopatz, A.; Lee, A.; Rokem, A.; Woods, C. N.; Fulton, C.; Masson, C.; Häggström, C.; Fitzgerald, C.; Nicholson, D. A.; Hagen, D. R.; Pasechnik, D. V.; Olivetti, E.; Martin, E.; Wieser, E.; Silva, F.; Lenders, F.; Wilhelm, F.; Young, G.; Price, G. A.; Ingold, G.-L.; Allen, G. E.; Lee, G. R.; Audren, H.; Probst, I.; Dietrich, J. P.; Silterra, J.; Webber, J. T.; Slavič, J.; Nothman, J.; Buchner, J.; Kulick, J.; Schönberger, J. L.; de Miranda Cardoso, J. V.; Reimer, J.; Harrington, J.; Rodríguez, J. L. C.; Nunez-Iglesias, J.; Kuczynski, J.; Tritz, K.; Thoma, M.; Newville, M.; Kümmerer, M.; Bolingbroke, M.; Tartre, M.; Pak, M.; Smith, N. J.; Nowaczyk, N.; Shebanov, N.; Pavlyk, O.; Brodtkorb, P. A.; Lee, P.; McGibbon, R. T.; Feldbauer, R.; Lewis, S.; Tygier, S.; Sievert, S.; Vigna, S.; Peterson, S.; More, S.; Pudlik, T.; Oshima, T.; Pingel, T. J.; Robitaille, T. P.; Spura, T.; Jones, T. R.; Cera, T.; Leslie, T.; Zito, T.; Krauss, T.; Upadhyay, U.; Halchenko, Y. O.; Vázquez-Baeza, Y. SciPy 1.0: Fundamental Algorithms for Scientific Computing in Python. *Nat. Methods* **2020**, *17* (3), 261–272. https://doi.org/10.1038/s41592-019-0686-2.

(4) Theret, D. P.; Levesque, M. J.; Sato, M.; Nerem, R. M.; Wheeler, L. T. The Application of a Homogeneous Half-Space Model in the Analysis of Endothelial Cell Micropipette Measurements. *J. Biomech. Eng.* **1988**, *110* (3), 190–199. https://doi.org/10.1115/1.3108430.
